# A taxonomic study of *Quercus langbianensis* complex based on morphology and DNA barcodes of classic and next generation sequences

**DOI:** 10.3897/phytokeys.95.21126

**Published:** 2018-02-07

**Authors:** Hoang Thi Binh, Nguyen Van Ngoc, Shuichiro Tagane, Hironori Toyama, Keiko Mase, Chika Mitsuyuki, Joeri Sergej Strijk, Yoshihisa Suyama, Tetsukazu Yahara

**Affiliations:** 1 Graduate School of Systems Life Sciences, Kyushu University, 744 Motooka, Fukuoka, 819-0395, Japan; 2 Department of Biology, Dalat University, 01 – Phu Dong Thien Vuong, Dalat, Vietnam; 3 Centre for Asian Conservation Ecology, Kyushu University, 744 Motooka, Fukuoka, 819-0395, Japan; 4 Kawatabi Field Science Centre, Graduate School of Agricultural Science, Tohoku University, 232-3 Yomogida, Naruko-onsen, Osaki, Miyagi 989-6711, Japan; 5 Biodiversity Genomics Team, Plant Ecophysiology & Evolution Group, Guangxi Key Laboratory of Forest Ecology and Conservation (under state evaluation status), College of Forestry, Daxuedonglu 100, Nanning, Guangxi, 530005, PR China; 6 State Key Laboratory for Conservation and Utilisation of Subtropical Agro-bioresources, College of Forestry, Guangxi University, Nanning, Guangxi 530005, PR China

**Keywords:** DNA barcoding, Fagaceae, MIG-seq, *Quercus*, taxonomy, Vietnam

## Abstract

The taxonomy of *Quercus langbianensis* and its relatives in Vietnam and Cambodia have been revised based on evidence obtained from field observations, morphological comparison of herbarium specimens and molecular analyses using both classic and next generation DNA markers. Based on Bayesian inference using *rbc*L, *mat*K and ITS regions and Neighbour-joining tree using genome-wide sequences amplified with multiplexed inter-simple sequence repeat (ISSR) primers (MIG-seq), the authors recognised ten species in the complex in Vietnam and Cambodia, three of which are newly described in this paper: *Q. baolamensis* **sp. nov.**, *Q. bidoupensis* **sp. nov.** and *Q. honbaensis* **sp. nov**. These new species are all phenotypically similar to *Q. langbianensis* *s. str.* in having lanceolate to oblanceolate leaf shape, upper 4–5/6–serrated leaf margin, acute or acuminate leaf apex and bracts of cupule arranged in 5–9 rings but distinguished both morphologically and phylogenetically. In molecular phylogenetic reconstructions, *Q. bidoupensis* is not close to any other species. In the Bayesian tree, *Q. honbaensis* is sister to both *Q. blaoensis* and *Q. camusiae* that are found in the same locality but morphologically distinct and those three species are sister to *Q. langbianensis* *s. str.*, while *Quercus baolamensis* is not sister to *Q. langbianensis* *s. str.* in both the Bayesian tree and MIG-seq tree. In addition, *Q. cambodiensis* and *Q. baniensis* previously reduced to *Q. langbianensis* *s. lat.* have been recognised as distinct species. Six species were in need of lectotypification and that is undertaken herein.

## Introduction

The genus *Quercus* L., with 400–500 species, is the largest genus in the family Fagaceae (Nixon 1993, Valencia-A et al. 2016). The genus is widely distributed in the northern Hemisphere including tropical montane forests in South East Asia and often dominant in temperate deciduous forests in East Asia, Europe and North America and desert scrubs in the Mediterranean (Nixon 1993, Hubert et al. 2014, Valencia-A et al. 2016). In Vietnam, 45 species of the genus *Quercus* have been recognised (Ban 2003, Ho 2003, Binh et al. in press) but taxonomic identities of some species remain to be revised. One of them is *Quercus langbianensis* Hickel & A.Camus (1921), described from Mt. Langbian of Lam Dong Province, southern Vietnam. Following previous studies including Deng et al. (2010), The Plant List (2013) adopted a broad concept of this species by treating the following seven names as synonyms: *Q. baniensis* A.Camus, *Q. blaoensis* A.Camus, *Q. camusiae* Trel. ex Hickel & A.Camus (a replacement name of *Q. geminata* Hickel & A.Camus), *Q. dilacerata* Hickel & A.Camus and *Q. donnaiensis* A.Camus from Vietnam, *Q. cambodiensis* Hickel & A.Camus from Cambodia and *Cyclobalanopsis faadoouensis* Hu from mainland of China. However, the authors’ recent comparison based on the collections of *Q. camusiae* and *Q. cambodiensis* from their type localities revealed that both *Q. camusiae* and *Q. cambodiensis* are distinct species from *Q. langbianensis* *s. str.* This finding triggered the re-examination of the taxonomy of *Q. langbianensis* *s. lat.* hereafter designated as “*Q. langbianensis* complex” and its similar species such as *Q. auricoma* A.Camus in which *Q. cambodiensis* was recently included (Tagane et al. 2017). Deng et al. (2010) studied the relationship of *Q. camusiae, Q. cambodiensis* and *Q. langbianensis* and concluded that the three species are phenotypically indistinguishable. However, their study was based on the comparison of a limited number of herbarium specimens.

In this study, specimens of the *Q. langbianensis* complex were observed and collected more widely: Mt. Hon Ba of Khanh Hoa Province (the type locality of *Q. camusiae*), some localities of Lam Dong Province (near the type locality of *Q. langbianensis* *s. str.*), Mt. Ba Na (the type locality of *Q. baniensis*) and Mt. Bokor of Cambodia (the type locality of *Q. cambodiensis*). In Mt. Hon Ba, *Q. camusiae* was found at the higher elevation whereas two additional morphologically similar but distinct species were found at the lower elevation. Observations in the field revealed that two neighbouring provinces of southern Vietnam, Khanh Hoa Province and Lam Dong Province, harbour the highest diversity of the *Q. langbianensis* complex including three unknown species. However, those species are phenotypically very similar to each other and evidence based on molecular analyses is needed to elucidate their identities and relationships.

Recently, molecular studies of the genus *Quercus* have succeeded in elucidating phylogenetic relationships within the genus by using multiple gene markers (Hubert et al. 2014, Simeone et al. 2016) or RAD-seq (Hipp et al. 2014, Cavender-Bares et al. 2015, Fitz-Gibbon et al. 2017). In this study, both classic multiple gene markers (*rbc*L, *mat*K and ITS) and genome-wide markers have been employed using the next generation sequencing platform (MIG-seq; Suyama and Matsuki 2015) to clarify relationships of the species within *Q. langbianensis* complex. As in RAD-seq, MIG-seq provides genetic markers of relatively short sequence reads determined by the next generation sequencer, but it is obtained with a PCR-based procedure without restriction enzyme digestion steps and is widely applicable to field samples even with low-quality DNA and/or small quantities of DNA (Suyama and Matsuki 2015).

The purpose of this paper is to revise the taxonomy of the *Q. langbianensis* complex based on evidence obtained from field observations, morphological studies and molecular data from both classic and next generation DNA markers. In conclusion, 10 species in the *Q. langbianensis* complex, including the seven species treated as synonyms of *Q. langbianensis* (The Plant List 2013) and the remaining three undescribed species have been distinguished. The latter three species are described as *Q. baolamensis*, sp. nov., *Q. bidoupensis* sp. nov. and *Q. honbaensis* sp. nov.

## Materials and methods

### Observations and collections in the field

**Figure 1.**
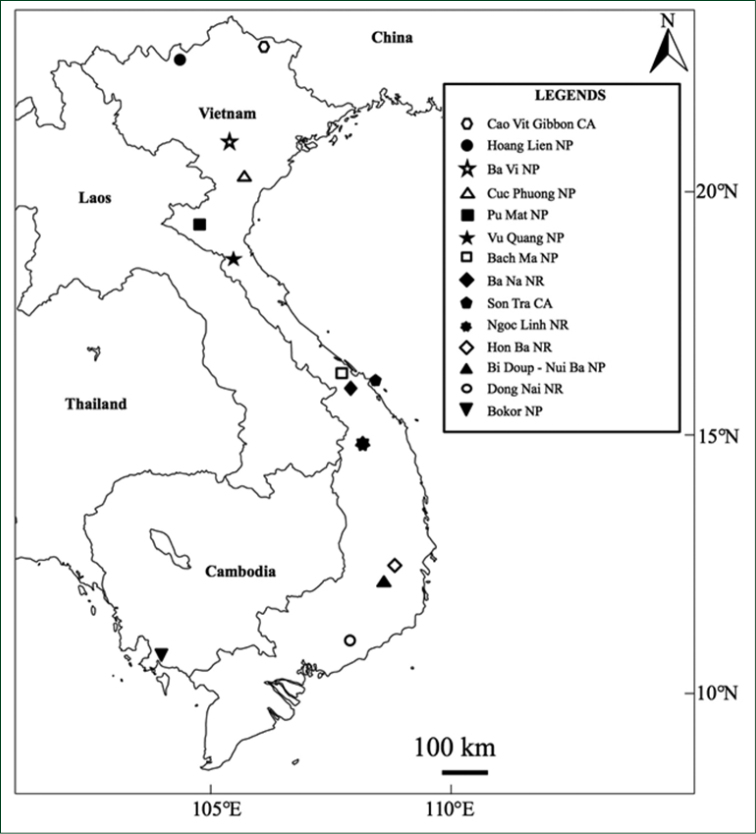
Collection sites in Vietnam and Cambodia in this study, including eight national parks, four nature reserves and two conservation areas.

The field surveys were carried out in 13 conservation areas (national parks, nature reserves and conservation areas) in Vietnam and one national park in Cambodia (Fig. 1). In Hon Ba Nature Reserve, Khanh Hoa Province, southern Vietnam, eight rectangular plots of 100 m × 5 m were placed at various locations from 225 m to 1,498 m altitude and girth and height for all the individual trees above 4 m tall within the plots were recorded (Table 1). The authors also recorded trees in the same way for the following localities: two plots at 1,553 m and 1,807 m in Bidoup-Nui Ba National Park, Lam Dong Province; three plots at 1,850 m, 2,225 m and 2,933 m in Hoang Lien National Park (Mt. Fan Si Pan), Lao Cai Province; four plots at 86 m, 650 m, 1,420 m and 1,720 m in Vu Quang National Park, Ha Tinh Province; two plots at 450 m and 1,274 m in Bach Ma National Park, Thua Thien Hue Province; and three plots at 833 m, 1,070 m and 1,376 m in Ngoc Linh Nature Reserve, Kon Tum Province. In these localities, general collections of vascular plants outside of the plots were also made, with particular attention to the species of Fagaceae. In addition to the above 6 conservation areas, general sampling of Fagaceae was undertaken in the following 6 conservation areas: Ba Na Nature Reverse, Da Nang Province; Ba Vi National Park, Ha Noi Capital; Cuc Phuong National Park, Ninh Binh Province; Dong Nai Nature Reserve, Dong Nai Province; Pu Mat National Park, Nghe An Province; Son Tra Conservation Area; and Cao Vit Gibbon Conservation Area, Cao Bang Province. In Mt. Bokor, Cambodia, 20 plots from 266 m to 1,048 m altitude were established and *Q. cambodiensis* was sampled (Zhang et al. 2016, Tagane et al. 2017). For each specimen collected, photographs were taken in the field and samples of silica gel-dried leaf pieces for DNA isolation were gathered.

**Table 1.** Altitudinal distribution of *Quercus* spp. found in Mt. Hon Ba.

Altitude (m)	*Quercus* trees found in each plot (tree height, girth)
1498	*Q. camusiae* (16 m, 88.5 cm)
1336	*Q. camusiae* (4.5 m, 4.8 cm)
1204	*Q. poilanei* (25 m, 86.5 cm; 25 m, 114 cm)
1021	No *Quercus* species
919	No *Quercus* species
617	*Quercus honbaensis* (12 m, 51.4 cm)
400	*Quercus honbaensis* (8 m, 19 cm; 18 m, 88 cm; 5 m, 10 cm)
225	*Quercus honbaensis* (4 m, 6.8 cm), *Q. blaoensis* (14 m, 50.5 cm; 11 m, 15.4 cm)

Amongst the collections of *Quercus*, the authors regarded species having the following traits as members of the *Q. langbianensis* complex: mature leaves are 12–17 cm long, 3–5 cm wide, serrated along the upper 5/6 to 1/3 margin (although young leaves of *Q. camusiae* are often almost entire), acute or acuminate at apex, cuneate at base and hairy when young but almost glabrous when mature; cupule obconical or bowl- or cup-shaped, bracts of cupule arranged in 5–9 rings and covers 1/4 to 2/3 of a nut that is ovoid or subglobose to ellipsoid. *Q. auricoma* in the *Q. langbianensis* complex was not included because mature leaves have entire margin and smaller size (5.5–7 cm long, 2–2.7 cm wide, from *E. Poilanei 13098* (P)).

In this study, 46 samples including 9 species of the *Quercus langbianensis* complex (*Q. baniensis, Q. baolamensis, Q. bidoupensis, Q. blaoensis, Q. cambodiensis, Q. camusiae, Q. donnaiensis, Q. honbaensis, Q. langbianensis* *s. str.*) and ten species of non-*Quercus langbiangensis* complex (*Q. annulata, Q. auricoma, Q. austrocochinchinensis, Q. braianensis, Q. djiringensis, Q. helferiana, Q. kerrii, Q. macrocalyx, Q. neglecta* and *Q. poilanei*) were used for morphological and DNA studies. One species of *Trigonobalanus*, *T. verticillatus* Forman was also analysed as an outgroup in phylogenetic analyses. Three to four sets of voucher specimens were collected from each locality and deposited in FU and herbaria of each protected area, DLU and VNM.

### DNA extraction

DNA was isolated from each silica-gel dried sample by the CTAB method (Doyle and Doyle 1987) with the following modifications: dried leaf material was milled by QIAGEN TissueLyser to obtain fine powder and washed three times in a 1 ml buffer (including 0.1 M HEPES, pH 8.0; 2% Mercaptoethanol; 1% PVP; 0.05 M Ascorbic acid) as in Toyama et al. (2015).

### Classic DNA sequencing

DNA regions of the large subunit of ribulose-1,5-biphosphate carboxylase oxygenase (*rbc*L), maturase K (*mat*K) and the internal transcribed spacer (ITS) were amplified with the following primer sets (sequence: 5’ to 3’): *rbc*La-F (ATGTCACCACAAACAGAGACTAAAGC, Levin et al. 2003), *rbc*L-724r (TCGCATGTACCTGCAGTAGC, Fay et al. 1997); *mat*K-XF (TAATTTACGATCAATTCATTC, Ford et al. 2009), *mat*K-1326R (TCTAGCACACGAAAGTCGAAGT, Cuénoud et al. 2002); ITS-18F (GTCCACTGAACCTTATCATTTAGAGG, Rohwer et al. 2009) and ITS-26R (GCCGTTACTAAGGGAATCCTTGTTAG, Rohwer et al. 2009). The sequences of *rbc*L, *mat*K and ITS were amplified with Tks GflexTM DNA Polymerase (Takara Bio, Kusatsu, Japan) following the protocols of Kress et al. (2009), Dunning and Savolainen (2010) and Rohwer et al. (2009), respectively. The PCR product was cleaned with 0.5 µl of ExoSap-IT enzyme (GE Healthcare, Little Chalfont, UK) and 1.5 µl of distilled water and incubated at 37°C for 30 min and subsequently at 80°C for 15 min for deactivation of the enzyme. Sequence reactions were continued using the ABI PRISM BigDye Terminator v3.1 Cycle Sequencing Kit (Applied Biosystems, Foster City, CA, USA). The reaction mixtures were analysed in an ABI 3730 automated sequencer (Applied Biosystems, Foster City, CA, USA).

### Next generation DNA sequencing – MIG-seq

For 105 samples, thousands of short sequences (loci) were amplified from each genome using primers designed for “multiplexed ISSR genotyping by sequencing” (MIG-seq, Suyama and Matsuki 2015) and presence/absence of each sequence (amplicon) were used in each sample for phylogenetic tree reconstruction regardless of whether it has SNP or not, as sequence-based dominant markers. The experimental standard conditions were performed following Suyama and Matsuki (2015). The 1st PCR step was performed to amplify ISSR regions from genomic DNA with MIG-seq primer set-1. The products of the 1st PCR were diluted 50 times for each 1st PCR product with deionised water. The 2nd PCR step was conducted independently to add individual indices to each sample using indexed primers. Then, 3 µl of each 2nd PCR product was pooled as a single mixture library. The mixture was purified and fragments in the size range 350-800 bp were selected by a Pippin Prep DNA size selection system (Sage Science, Beverly, MA, USA). Finally, the concentration of size-selected library was measured by using a SYBR green quantitative PCR assay (Library Quantification Kit; Clontech Laboratories, Mountain View, CA, USA) with approximately 10 pM of libraries that were used for sequencing on an Illumina MiSeq Sequencer (Illumina, San Diego, CA, USA), using a MiSeq Reagent Kit v3 (150 cycle, Illumina).

### Phylogenetic analyses

For classical phylogenetic analyses, a phylogenetic tree was constructed by combining nucleotide sequences of the three DNA regions comprising *rbc*L, *mat*K and ITS for 30 samples of 29 *Quercus* species and one *Trigonobalanus verticillatus* (as an outgroup). All DNA sequences were newly generated in this study. The sequences were aligned by MEGA v7.0 (Kumar et al. 2016). For reconstructing phylogeny, a Bayesian method implemented in the programme BEAST v1.8.4 (Drummond et al. 2012) was used. The GTR + γ model of molecular evolution and an uncorrelated lognormal (UCLN) relaxed-clock model were selected to infer relative divergence times. In computation, the programme was started with a random tree and a tree prior that was useful for species-level was set according to a Yule process (Drummond and Rambaut 2007). Five independent chains of 100 million generations each were run with sampling every 10,000 generations. The first 1,000 trees were discarded as burn-in from each run. The remaining trees from each run were combined by using LogCombiner v 1.6.1 (Drummond and Rambaut 2007). Amongst the posterior distribution of 9,000 trees, the maximum clade credibility tree was identified using TreeAnnotator v 1.6.1 (Drummond and Rambaut 2007) with a posterior probability limit of 0.5 and median node heights. The congruence amongst *rbc*L, *mat*K and ITS trees was tested using the incongruence length difference test (Farris et al. 1994) implemented in PAUP* 4.0b10 (Swofford 2003). As the incongruence was rejected (p=0.06), a combined tree using concatenated sequences was constructed.

For MIG-seq, raw data were pretreated from 105 samples and quality control was completed following Suyama and Matsuki (2015). The programme ‘fastx_trimmer’ in the FASTX-Toolkit (http://hannonlab.cshl.edu/fastx_toolkit/) was used to trim read 2 sequences including 12 bases of SSR region and two bases of anchor sequences in the 1st primers. The authors used option ‘quality_ filter’ of FASTX-Toolkit to select reads in which 40% or more sequences had quality scores Q30 or more. Then the TagDust programme (Lassmann et al. 2009) was used to remove the reads derived from extremely short library entries and to trim read 1 and read 2 sequences. Then, loci were assembled from the quality-filtered reads data with the *de novo* map pipelines (ustacks, cstacks, sstacks) in Stacks software package version 1.35 (Catchen et al. 2011) and then a table prepared of presence/absence of loci in each individual from the outputs of the populations pipeline of Stacks 1.35. Using ustacks, homologous sequences (loci) were assembled in each individual with the following settings: minimum depth of coverage (m) = 10, maximum distance allowed between stacks (M) = 1, maximum distance allowed to align secondary reads to primary stacks (N) = 1 and maximum gaps = 2. Using cstacks, a catalogue of consensus loci was built for all the individuals by assembling loci in each individual assembled using ustacks, with the number of mismatches allowed between sample loci (n) = 2. Using sstacks, IDs of loci were associated in each individual with IDs of the consensus loci. Finally, presence/absence of loci were determined in each individual from a haplotypes list obtained using the populations pipeline. The populations pipeline output file haplotypes.tsv provides genotypes of individuals at each locus. For each individual, the authors recorded a locus that had genotype information as “1” and a locus that had no genotype information as “0”. The authors obtained a list of loci that were detected in at least one individual (1/105 = 0.01) with the following settings: all samples belong to the same population and threshold frequency of haplotype count in a population (r) = 0.001, a threshold one-order higher than 0.01. Using presence/absence (1/0) data of loci, the authors computed distance matrix, constructed a neighbour-joining (NJ) tree and examined the reliability of tree topology by bootstrapping with 1000 replicate using PHYLIP ver. 3.695 (Shimada and Nishida 2017) as follows; 1000 times re-sampling with Seqboot, distance computation with Restdist, tree construction with Neighbour and consensus tree construction with Censense. The resulted tree was visualised with FigTree v1.4.3 (http://tree.bio.ed.ac.uk/software/figtree/). A phylogenetic analyses was first made for 105 samples including more *Quercus* species and then the sample size reduced to 31 by focusing on the *Q. langbianensis* complex. A total of 16,809 loci were used for the final phylogenetic tree.

### Morphological and taxonomic comparison

The collections contain considerable numbers of sterile specimens including those from young trees that are often morphologically different from adult trees. Thus, after phylogenetic trees were obtained, morphological traits of leaves and shoots were carefully re-examined as well as reproductive organs if available and species were distinguished. If two OTUs are morphologically distinguishable and also not monophyletic on phylogenetic trees, these were regarded as two distinct species. Then, these were identified by a thorough literature review and comparisons with type specimen images available online (e.g. JSTOR Global Plants, http://plants.jstor.org/). In *Q. langbianensis* complex, lectotypification was needed for *Q. baniensis, Q. blaoensis, Q. cambodiensis, Q. camusiae, Q. dilacerata* and *Q. donnaiensis*. One of the co-authors, J.S. Strijk, examined specimens at P for lectotypification; selected for each species, was one of the specimens cited in the original description, which best represents the diagnostic traits of each species.

## Results

### Observation in the field

In Hon Ba Nature Reverse, tree diversity was examined in eight plots of 100 m × 5 m and four species of *Quercus* (Table 1) were found including *Q. poilanei* and three species of the *Q. langbianensis* complex: *Q. blaoensis*, *Q. camusiae* and an undescribed species, *Q. honbaensis*. *Quercus camusiae* was found in the two plots at 1,336 m and 1,498 m altitude and one of canopy trees in the latter. *Quercus honbaensis* was found in three plots at 225 m, 400 m and 617 m altitude and occurred sympatrically with *Q. blaoensis* in the plot at 225 m altitude. *Quercus honbaensis* was one of the canopy trees at both 225 m and 400 m altitude (Table 1). In late February of 2014, *Q. honbaensis* had mature fruits and *Q. blaoensis* had young fruits. Two species were distinct in pubescence on young shoots (*Q. honbaensis* has long, very thin and curly hairs vs. *Q. blaoensis* has short, thicker and straight hairs). *Quercus camusiae* was distinct from *Q. honbaensis* and *Q. blaoensis* in that shoots and leaves were golden tomentose when young.

In Bidoup-Nui Ba National Park, approximately 100 km west of Mt. Hon Ba, tree diversity was examined in two plots at 1,553 m and 1,807 m altitude and *Q. langbianensis* *s. str.* was found at 1,553 m altitude. *Quercus langbianensis* *s. str.* was similar to *Q. camusiae* in having golden tomentose cupules, but different in distinctly toothed leaves and longer nuts (vs. almost entire or with only a few low teeth in *Q. camusiae*). The flora was surveyed above 800 m altitude in Bidoup-Nui Ba National Park and *Q. camusiae* and *Q. honbaensis* were not found. On the other hand, two additional and unknown species of the *Q. langbianensis* complex were found: *Q. bidoupensis* and *Q. donnaiensis*. *Quercus bidoupensis* was distinct from *Q. langbianensis* *s. str.* in having oblong-lanceolate leaves, acuminate and slightly caudate at apex and undulate and distinctly serrate along the upper half of the margin. *Quercus donnaiensis* was similar to *Q. bidoupensis* in leaf shape but differs in its margin not being undulate, serrated only near the apex and with 3-5 teeth. From the general collection in Lam Dong Province, three species of the *Q. langbianensis* complex were collected: *Q. bidoupensis* and *Q. donnaiensis* in Lam Tranh District and another undescribed species, *Q. baolamensis*, in Bao Lam District.

In Ba Na Nature Reserve and Son Tra Natural Conservation Area, central Vietnam, *Q. baniensis* of the *Q. langbianensis* complex and *Q. poilanei* and *Q. auricoma* of non-*Q. langbianensis* complex were found.

In the top plateau of Mt. Bokor, Cambodia, *Q. cambodiensis* of the *Q. langbianensis* complex and *Q. augustinii* of non-*Q. langbianensis* complex were collected.

### A phylogenetic tree combining three DNA regions (*rbcL*, *matK*, and *ITS*)

**Figure 2.**
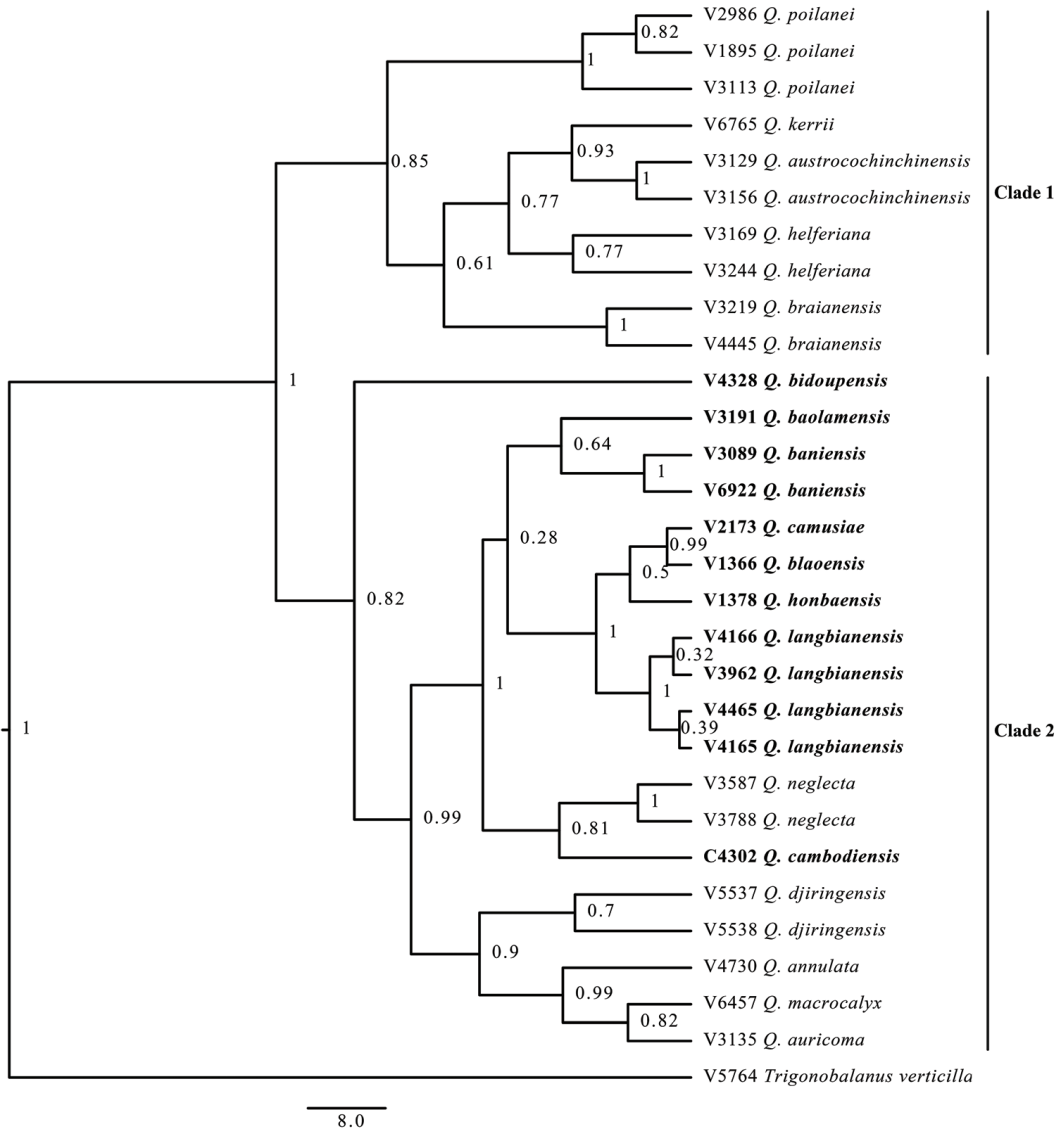
Bayesian phylogeny of 29 samples of *Quercus* and one *Trigonobalanus* (outgroup) based on *rbc*L, *mat*K and ITS sequences. Braches are labelled with posterior probabilities.

A total of 2,034 bases consisting of three DNA regions (657 bp for *rbc*L, 834 bp for *mat*K and 543 bp for ITS) included 142 variable sites, amongst which 56 bases were parsimony-informative (Table 2). According to the Bayesian tree combining the three regions (Fig. 2), two major clades were supported by posterior probabilities higher than 80%: Clade 1 with 85 % posterior probability consists of five species of non-*Quercus langbianensis* complex (*Q. poilanei*, *Q. kerrii*, *Q. austrocochinchinensis*, *Q. helferiana* and *Q. braianensis*) and Clade 2 with 82 % posterior probability including seven species of the *Q. langbianensis* complex (*Q. cambodiensis* and six Vietnamese species) and five species of non-*Quercus langbianensis* complex (*Q. neglecta* nested with the *Q. langbianensis* complex and *Q. annulata*, *Q. auricoma*, *Q. djiringensis* and *Q. macrocalyx*). In Clade 2, *Q. cambodiensis* was sister to *Q. neglecta* with 81% posterior probability and clearly separated from the Vietnamese species of the *Q. langbianensis* complex (*Q. langbianensis* *s. str.*, *Q. baniensis*, *Q. blaoensis*, *Q. honbaensis*, *Q. baolamensis* and *Q. camusiae*). *Quercus langbianensis* *s. str.* was sister to *Q. blaoensis*, *Q. camusiae* and *Q. honbaensis* with a strong branch support (PP = 1.00). *Quercus camusiae* was sister to *Q. blaoensis* with a high branch support (PP = 0.99). *Quercus baolamensis* and *Q. baniensis* were clustered together, but with weak branch support (PP = 0.64).

**Table 2.** Summary statistics of datasets used for phylogenetic inference comprising *rbc*L, *mat*K and ITS sequences.

Regions	*rbc*L	*mat*K	ITS	Combined data
Aligned sequence length	657	834	543	2034
Variable DNA sites	9	35	98	142
Parsimony-informative sites	3	9	44	56

Trees based on single gene sequences gave lower resolution but the ITS tree (see Suppl. material 1: Figure 1) supported the following points: (1) A clade, consisting of seven species of the *Q. langbianensis* complex and *Q. neglecta*, was supported by 100 % PP. (2) *Quercus bidoupensis* was not clustered with the other seven species of the *Q. langbianensis* complex. (3) A clade, including five species of non-*Q. langbianensis* complex, was supported by 70 % PP. In the cpDNA tree (see Suppl. material 1: Figure 2), neither the seven species of the *Q. langbianensis* complex nor the five species of non-*Q. langbianensis* complex was monophyletic. Neither *Q. poilanei*, *Q. austrocochinchinensis*, *Q. helferiana* and *Q. braianensis* was monophyletic whereas those four species were monophyletic in the ITS tree.

### A phylogenetic tree using MIG-seq

**Figure 3.**
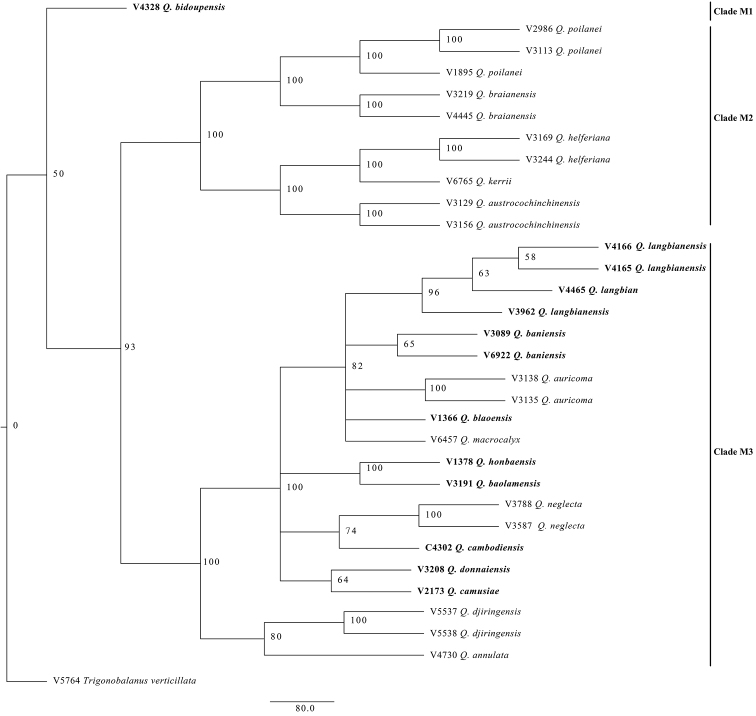
NJ tree of 31 samples of *Quercus* and one *Trigonobalanus* (outgroup) based on presence/absence data of 16,809 MIG-seq loci. Branches are labelled with bootstrap supports (% of 1000 replicates).

A neighbour-joining (NJ) tree based on MIG-seq for 31 samples of *Quercus* recognised three major clades excluding an outgroup of *Trigonobalanus* (Fig. 3). Clade M1 includes single species, *Q. bidoupensis.* Clade M2 with a 100 % bootstrap value consists of five species of non-*Q. langbianensis* complex (*Q. poilanei, Q. kerrii, Q. austrocochinchinensis, Q. helferiana* and *Q. braianensis*). Clade M3 with 100 % bootstrap value includes *Q. neglecta*, *Q. macrocalyx*, *Q. auricoma* and eight species of the *Q. langbianensis* complex. Within this clade, *Q. cambodiensis* was sister to *Q. neglecta* with a 74 % bootstrap value. *Quercus honbaensis* and *Q. baolamensis* were monophyletic with a bootstrap value of 100 %. *Quercus donnaienis* and *Q. camusiae* were also monophyletic with a bootstrap value of 75 %. *Quercus blaoensis* and *Q. langbianensis* *s. str.* of the *Q. langbianensis* complex are clustered with *Q. baniensis, Q. auricoma, Q. macrocalyx*, forming a clade with 82 % bootstrap value.

## Discussion

**Figure 4.**
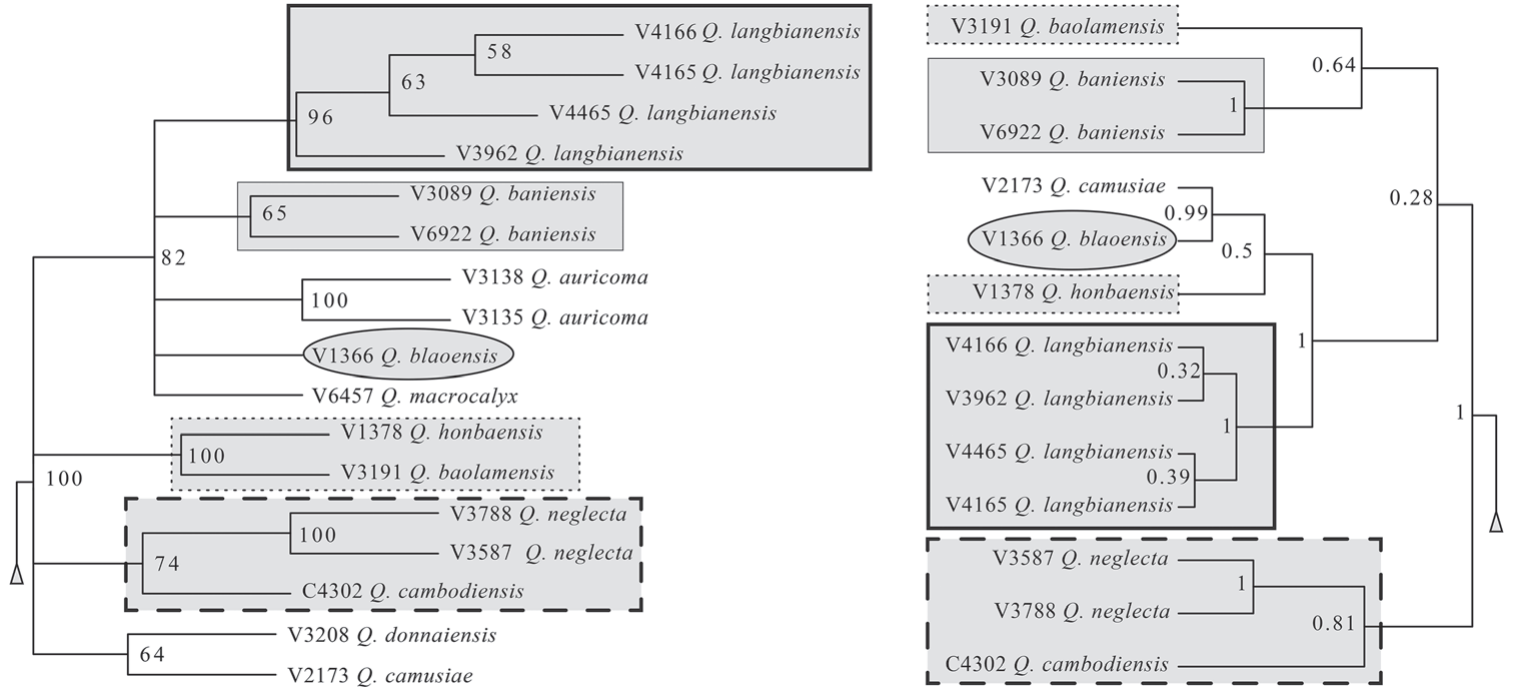
Comparison of *Q. langbianensis* complex between NJ tree (left, Clade M3 of Fig. 3) and Bayesian tree (right: Clade 2 of Fig. 2).

The results of the three gene tree (Bayesian tree) and MIG-seq tree (NJ tree) were mostly consistent. First, five species of non-*Q. langbianensis* complex (*Q. poilanei, Q. kerrii, Q. austrocochinchinensis, Q. helferiana* and *Q. braianensis*) formed a highly supported clade, Clade 1 or Clade M2. This clade was supported also in the ITS tree. Second, three gene and MIG-seq trees matched in Clade 2 and Clade M3. Third, the species of the *Q. langbianensis* complex except *Q. bidoupensis* formed a highly supported clade (also in ITS tree) and *Q. auricoma, Q. macrocalyx* and *Q. neglecta* of non-*Q. langbianensis* complex were included in this clade (Fig. 4). Fourth, *Q. cambodiensis* was sister to *Q. neglecta* and separated from the Vietnamese species of the *Q. langbianensis* complex by relatively high supports (posterior probability 0.81 and bootstrap probability 74%). Fifth, *Q. bidoupensis* was placed in Clade 2 or Clade M1 and not close to the other species of the *Q. langbianensis* complex (also in ITS tree). The cpDNA tree did not support monophilies of *Q. poilanei*, *Q. austrocochinchinensis, Q. helferiana* and *Q. braianensis* that were monophyletic in the ITS tree, three gene tree and MIG-seq tree and thus the cpDNA tree alone provides less reliable evidence.

The consistent topology of three gene and MIG-seq trees (Figs 2–4) provided reliable evidence to resolve taxonomy of “species” currently treated as synonyms of *Q. langbianensis* s. lat. (*Q. camusiae*, *Q. blaoensis*, *Q. cambodiensis* and *Q. baniensis*). First, *Q. cambodiensis* is separated as a species because it is sister to *Q. neglecta* that is morphologically distinct in linear leaves and small nuts and has been treated as a distinct species in the Flora of China (Huang et al. 1999 as *Cyclobalanopsis neglecta*). Amongst the others, both *Q. camusiae* and *Q. blaoensis* are native in the Hon Ba Nature Reserve where *Q. camusiae* occurs at the higher elevation and *Q. blaoensis* occurs at the lower elevation (Table 1). As *Q. camusiae* and *Q. blaoensis* are sister to each other in the MIG-seq tree and not sympatric but paratactic in the distribution, those can be treated as two infraspecific taxa (varieties or subspecies) or two different species. Considering the morphological distinction described above, the latter treatment has been adopted. *Quercus blaoensis* co-occurs with another undescribed species: *Q. honbaensis*. As *Q. blaoensis* and *Q. honbaensis* are sympatric and morphologically distinct, those are recognised as different species. The monophyly of *Q. honbaensis* and *Q. baolamensis* was strongly supported in the MIG-seq tree with a bootstrap value of 100%. While *Q. honbaensis* occurs at an elevation lower than 617 m in Hon Ba Nature Reserve, Khanh Hoa Province, *Q. baolamensis* is collected at 1000 m from the Lam Dong province. Considering this distinction in cupule and nut morphology (Figs 6, 12), they are treated as two distinct species. While *Q. baniensis* is found in Da Nang Province of central Vietnam, the other five species (*Q. baolamensis, Q. blaoensis, Q. camusiae, Q. honbaensis* and *Q. langbianensis* *s. str.*) occur in Khanh Hoa or Lam Dong Province of southern Vietnam. From phylogenetic trees and morphological observations, it is difficult to relate *Q. baniensis* to any of the five species. In particular, a sister relationship between *Q. langbianensis* *s. str.* and *Q. baniensis* is not strongly supported (Fig. 4). Thus, *Q. baniensis* is treated as a distinct species.

Although the topologies of the three gene and MIG-seq tree are mostly consistent, there are some notable differences, particularly in Clade 2 and Clade M3 containing the *Q. langbianensis* complex (Fig. 4). In the Bayesian tree, based on the three regions of *rbc*L, *mat*K and ITS, the monophyly of the Vietnamese species of the *Q. langbianensis* complex was only weakly supported (PP = 0.28), whereas it was strongly supported in MIG-seq tree (bootstrap value 100 %). This higher support in MIG-seq tree was obtained because MIG-seq provided more informative sites for constructing phylogenetic relationships amongst the species in the *Q. langbianensis* complex. *Quercus auricoma* and *Q. macrocalyx* were included in a clade of the *langbianensis* complex in the MIG-seq tree but clustered with *Q. annulata* in the Bayesian tree. Further studies using more gene markers are needed to derive a conclusion on the placement of these two species.

A comparison, based on morphological characters both in the field and from dried specimens of the herbarium and the molecular evidence for the *Q. langbianensis* complex, revealed that *Q. baniensis*, *Q, blaoensis*, *Q. cambodiensis*, *Q. camusiae* and *Q langbianensis* *s. str.* are all distinct species (Table 3). In addition, it is concluded that three species amongst the *Q. langbianensis* complex are undescribed and below they are described as *Q. baolamensis*, *Q. bidoupensis* and *Q. honbaensis.* In this study, only sterile specimens of *Q. donnaiensis* were collected and the sequence of ITS for *Q. donnaienis* could not be determined due to low DNA quality. In the MIG-seq tree, *Q. donnaienis* and *Q. camusiae* were monophyletic with a bootstrap value of 64 %. In vegetative traits, *Q. donnaiensis* is distinguished from *Q. camusiae* in having distinct serrations on the upper 1/3 of the leaf margin (vs. almost entire in *Q. camusiae*). Additional materials having fruits need to be examined to conclude whether those two are distinct species or infraspecific taxa. In the following taxonomic section, they are tentatively treated as two species. Amongst species treated as synonyms of *Q. langbianensis* *s. lat.* (Plant List 2013), *Q. dilacerata* is morphologically distinct as described in the following taxonomic section, but DNA samples of this species could not be obtained. Further studies using phylogenetic analyses are required to clarify the identity of *Q. dilacerata*.

**Table 3.** Morphological comparison of *Quercus langbianensis* complex.

Characters	*Q. bidoupensis*	*Q. camusiae*	*Q. cambodiensis*	*Q. baniensis*	*Q. honbaensis*	*Q. dilacerata*	*Q. blaoensis*	*Q. donnaiensis*	*Q. baolamensis*	*Q. langbianensis*
Young shoot	Almost glabrous	Golden tomentose			Curly hairy	Golden tomentose	Straight hairy	Golden tomentose	Almost glabrous	
Leaf margin	Undulate, distinctly serrate in upper 1/2	Not undulate, almost entire or with a few low teeth in upper 1/4		Not undulate, distinctly serrate in upper 1/3	Not undulate, distinctly serrate in upper 5/6–3/4(–2/3)	Not undulate, distinctly serrate in upper 1/3			Not undulate, distinctly serrate in upper 1/2	Not undulate, distinctly serrate in upper 1/3
Length of petioles	1.3–2 cm	1–1.6 cm	1–2.2 cm	1.2–2(–2.9) cm	0.8–1 cm	1–1.4 cm	0.9–1.8 cm	1–2 cm	0.4–1 cm	1–2 cm
Number of secondary veins	10–13 pairs	8–13 pairs	7–11 pairs		(9–)10–14(–16) pairs	12–14 pairs	8–13 pairs	9–12(–14) pairs	(7–)10–13 pairs	10–12 pairs
Cupule shape	Obconical	Cup-shaped		Obconical		Bowl-shaped	Cup-shaped			
Cupule coverage	Enclosing 1/3 of the nut	Enclosing <1/2 of the nut		Enclosing 2/3 of the nut	Enclosing 1/3–1/2 of the nut	Enclosing 2/3 of the nut		Enclosing 1/2 of the nut	Enclosing 1/3 of the nut	
Cupule bract	5–6 rings	6 rings	7–8 rings	6–8 rings				5–6 rings	6–9 rings	
Cupule bract margin	Entire	Sparsely dissected in the lower rings	Distinctly toothed in two lower rings	Undulate		Distinctly toothed in all rings	Nearly entire, not undulate			
Nut shape	Ovoid	Subglobose		Ovoid	Obovoid to ellipsoid	Subglobose	Ovoid	Subglobose	Ovoid-ellipsoid	Obovoid to ellipsoid
Nut scar	Convex		Flat	Convex				Convex	Flat	Convex
Nut hairiness	Glabrous	Densely hairy		Sparsely hairy			Densely hairy	Sparsely hairy		Densely hairy

While *Q. cambodiensis* is treated as a synonym of *Q. auricoma* by Tagane et al. (2017), those two species are not sister to each other in both Bayesian and MIG-seq trees. The treatment of Tagane et al. (2017) is based on the broad concept of *Q. auricoma* adopted in the Flora of Thailand (Phengklai 2008) in which a species morphologically similar to *Q. cambodiensis* in northern and north-eastern Thailand is treated as *Q. auricoma*. However, after examining the collection of *Q. auricoma* from Son Tra (*V3135, V3138*) that is morphologically identical with the species of the type specimen of *Q. auricoma*, it is concluded that the species treated as *Q. auricoma* in the Flora of Thailand (Phengklai 2008) is different from genuine *Q. auricoma*, in that leaves are serrate along the upper 1/2–1/3 margin (vs. completely entire in *Q. auricoma*), nuts ovoid to oblong (vs. suborbicular) and cupules densely hairy (vs. less hairy). As far as is known, *Q. auricoma* is endemic to Vietnam. Further studies are needed to elucidate the identity of the species called “*Q. auricoma*” in Thailand.

### Key to the species of *Quercus langbianensis* complex in Vietnam and Cambodia

**Table:** 

1	Leaves undulate, distinctly serrate in the upper 1/2. Cupules obconical, enclosing 1/3 of the nut, bracts set in 5–6 rings, margin entire. Nut ovoid, scar convex	***Q. bidoupensis***
–	Leaves not undulate	**2**
2	Leaves almost entire or with a few low teeth. Cupule cup-shaped, enclosing <1/2 of nut, bracts set in 6–8 rings, margin undulate at least in the lower rings. Nut subglobose, scar convex or flat	**3**
–	Leaves distinctly serrate in the upper 5/6 to 1/3 of margin	**4**
3	Cupule distinctly narrowed at base, bracts set in 6 rings, sparsely dissected in the lower rings. Nut scar convex	***Q. camusiae***
–	Cupule not distinctly narrowed at base, bracts set in 7–8 rings, margin distinctly toothed in two lower rings. Nut scar flat	***Q. cambodiensis***
4	Cupule obconical	**5**
–	Cupule cup-shaped or bowl-shaped	**6**
5	Margin distinctly serrate in the upper 1/3; secondary veins 7–11 pairs; petioles 1.2–2(–2.9) cm long; Cupules enclosing 2/3 of the nut. Nut ovoid	***Q. baniensis***
–	Margin distinctly serrate in the upper 5/6–3/4(–2/3); secondary veins (9–)10–14(–16) pairs; petioles 0.8–1 cm long. Cupules enclosing 1/3–1/2 of the nut. Nut obovoid to ellipsoid	***Q. honbaensis***
6	Cupule bowl-shaped, enclosing about 2/3 of nut, bracts set in 7 rings, bract margin distinctly toothed in all rings. Nut subglobose	***Q. dilacerata***
–	Cupule cup-shaped, enclosing 1/3–2/3 of nut, bracts set in 5–9 rings, bract margin nearly entire	**7**
7	Cupules enclosing 2/3 of the nut. Nut ovoid. Young shoots covered with straight whitish hairy. Leaves distinctly serrate in the upper 1/3	***Q. blaoensis***
–	Cupules enclosing 1/3–1/2 of the nut. Nut subglobose, ovoid-ellipsoid, obovoid to ellipsoid. Young shoots covered with golden tomenose or almost glabrous. Leaves regularly distinctly serrate in the upper 1/3–1/2	**8**
8	Cupules enclosing 1/2 of the nut, bracts set in 5–6 rings. Leaves regularly distinctly serrate in the upper 1/3. Nut subglobose, scar convex	***Q. donnaiensis***
–	Cupules enclosing 1/3 of the nut, bracts set in 6–9 rings. Leaves regularly distinctly serrate in the upper 1/2 or upper 1/3. Nut scar flat or convex	**9**
9	Leave regularly distinctly serrate in the upper 1/2; petiole 0.4–1 cm long. Nut ovoid–ellipsoid, scar flat, sparsely hairy	***Q. baolamensis***
–	Leave regularly distinctly serrate in the upper 1/3; petiole 1–2 cm long. Nut obovoid to ellipsoid, scar convex, densely hairy	***Q. langbianensiss. str.***

## Taxonomic treatments of *Quercus langbianensis* complex in Vietnam and Cambodia

### Quercus baniensis

Taxon classificationPlantaeFagalesFagaceae

A.Camus

Fig. 5

Quercus baniensis A.Camus, Chênes Atlas 2: 123, pl. 231 (1935–1936), nom. nud.; Bull. Soc. Bot. France 83: 343 (1936).

#### Type.

VIETNAM. “Mont Bani, in the main coast range about 25 kilometres from Tourane”, 4–13 June, 1927, *J. & M.S. Clemens 3455* (lectotype: P [P00753998!]; isolectotype: BM [BM000839274, BM000839274, image!], MICH [MICH1210512, image!], U [U0238780, image!], US [US00089422, image!], designated here).

**Figure 5.**
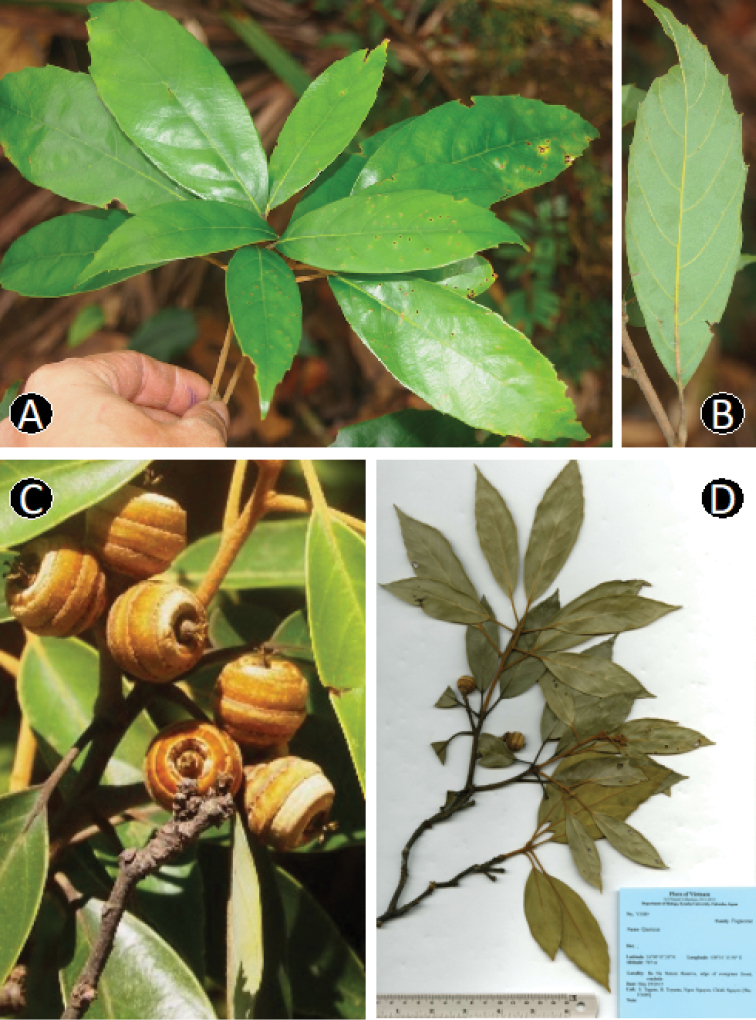
*Quercus baniensis* A.Camus. **A** Leafy twig **B** Abaxial side of mature leaf **C** Infructescence and young fruits **D** Dried specimen. Materials: **A, B** from *Hoang T.S. & Tagane S. V6922* **C, D** from *Tagane et al. V3089*.

#### Distribution and habitat.

VIETNAM. Da Nang Province: Ba Na Nature Reserve. In this study, this species was found along the roadside and edge of evergreen forest, at 707 and 789 m altitude.

#### Additional specimens examined.

VIETNAM. Ba Na Nature Reserve, 16°00'07.30"N, 108°01'33.90"E, alt. 707 m, 29 May 2015, *Tagane S., Toyama H., Nguyen N., Nguyen C. V3089* [young fr.] (DLU, FU); ibid., 16°00'10.0"N, 108°02'17.8"E, alt. 789 m, 19 Feb. 2017, *Hoang T.S. & Tagane S. V6922* (DLU, FU).

#### Note.

Camus (1935) illustrated *Quercus baniensis* in Chênes Atlas 2 (Pl.231) and later Camus (1936) effectively described this species based on the specimen *Clemens 3455* collected from mountain Bani, Vietnam. The authors examined six specimens of *Clemens 3455* in P, BM, MICH, U and US directly or by using digitised images on the web. Amongst them, *Clemens 3455* in P [P00753998] was selected as the lectotype of *Q. baniensis* because the trait of a nut is well represented in this specimen.

### Quercus baolamensis

Taxon classificationPlantaeFagalesFagaceae

Binh & Ngoc sp. nov.

urn:lsid:ipni.org:names:77175738-1

Fig. 6

#### Diagnosis.

*Quercus baolamensis* is most similar to *Q. langbianensis* *s. str.*, but differs in having the leaf margin regularly distinctly serrate in the upper 1/2 (vs. serrate in the upper 1/3) and shorter petioles 0.4–1 cm long (vs. 1–1.8 cm long).

**Figure 6.**
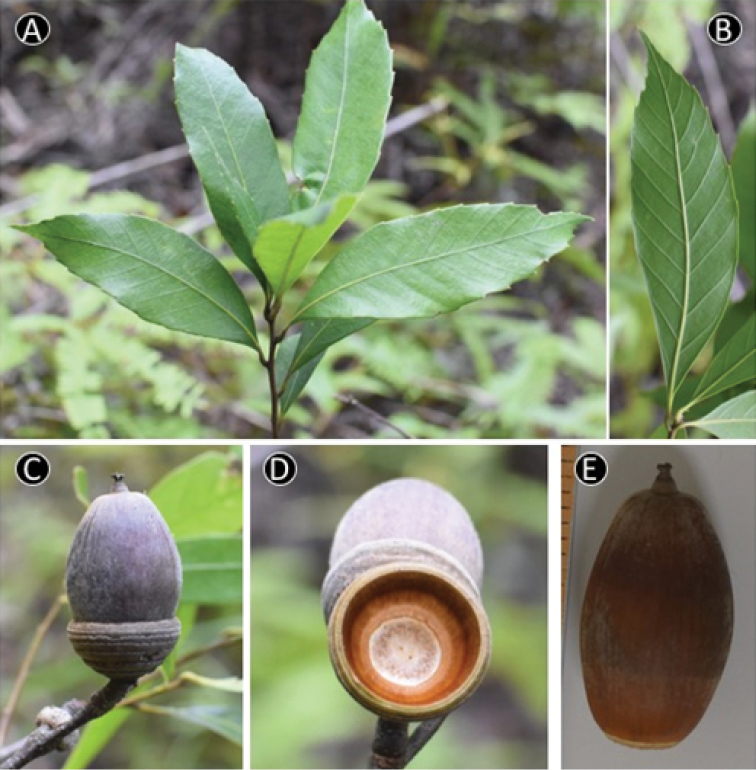
*Quercus baolamensis* Binh & Ngoc. **A** Leafy twig **B** Abaxial side of mature leaf **C** Mature fruit **D** Inside of cupule **E** Nut. Materials: **A–E** from *Ngoc et al. V3191*.

#### Type.

VIETNAM. Lam Dong Province: Bao Lam District, B40 Pass, roadside and edge of evergreen forest, 11°43'37"N, 107°42'34.5"E, alt. 1,000 m, 13 June 2015, with fruits, *Ngoc N.V., Binh H.T., Dung L.V., Truong N.K. V3191* (holotype: KYO!; isotypes: FU!).

#### Description.

Tree, 6–8 m tall. Young twigs almost glabrous except near bud, 1–1.2 mm in diam., sometimes sulcate. Old twigs glabrous, brownish-black when dry, lenticellate. Stipules linear, 3–5 mm long, hairy on both surfaces, caducous. Leaf blades elliptic to elliptic-lanceolate or rarely oblanceolate, (5.2–)9–15 × 1.7–4.5 cm, thinly coriaceous, glossy adaxially, pale green abaxially, acuminate at apex, cuneate at base, margin regularly distinctly serrate in the upper 1/2, having 9–12 teeth per side, glabrous on both surfaces; midrib slightly prominent adaxially, prominent abaxially, lateral veins (7–)10–13 pairs, straight and running into the teeth of margin, slightly prominent adaxially, prominent abaxially, at an angle of 40–45 degrees from midrib, tertiary veins scalariform-reticulate, visible on both surfaces; petioles 0.4–1 cm long, whitish hairy when young, glabrescent. Male and female inflorescences not seen. Infructescences axillary or terminal, erect spike, rachis 0.5–1.4 cm long, 1–3 mm in diam., tomentose when young, glabrescent when mature. Mature fruits ca. 2.9 cm high (including cupule), usually 1 (or 2) per infructescence, sessile; cupules obconical, 1.2 cm high, 1.5 cm in diam., enclosing 1/3 of the nut, wall comprising bracts, arranged in 7 rings, margin of rings nearly entire; nut ovoid-ellipsoid, 2.5 cm high, 1.5 cm in diam., apex nearly flat, sparsely hairy except densely appressed hairy around stylopodia, stylopodia up to 4 mm long, basal scar flat, 0.8 cm in diam., glabrous.

#### Phenology.

Fruiting specimens were collected in June.

#### Distribution and habitat.

VIETNAM. Lam Dong Province: Bao Lam District. At present, this species is known only from the type locality. Only one individual was found along the roadside and edge of evergreen forest, at 1,000 m altitude.

#### Etymology.

The specific epithet is derived from the name of its type locality, Bao Lam District.

### Quercus bidoupensis

Taxon classificationPlantaeFagalesFagaceae

Binh & Ngoc sp. nov.

urn:lsid:ipni.org:names:77175739-1

Fig. 7

#### Diagnosis.

Similar to *Quercus langbianensis* *s. str.* in leaf shape, the number of secondary veins and basal scar of the nut convex, but distinguished in having bud oblong to ellipsoid (vs. globose to broadly ovoid), undulate and distinctly serrate leaf margin along the upper half (vs. regularly distinctly serrate in the upper 1/3), obconical cupules (vs. cup-shape), bracts of cupule arranged in 5–6 rings (vs. 6–9 rings), and nut ovoid (vs. obovoid to ellipsoid).

**Figure 7.**
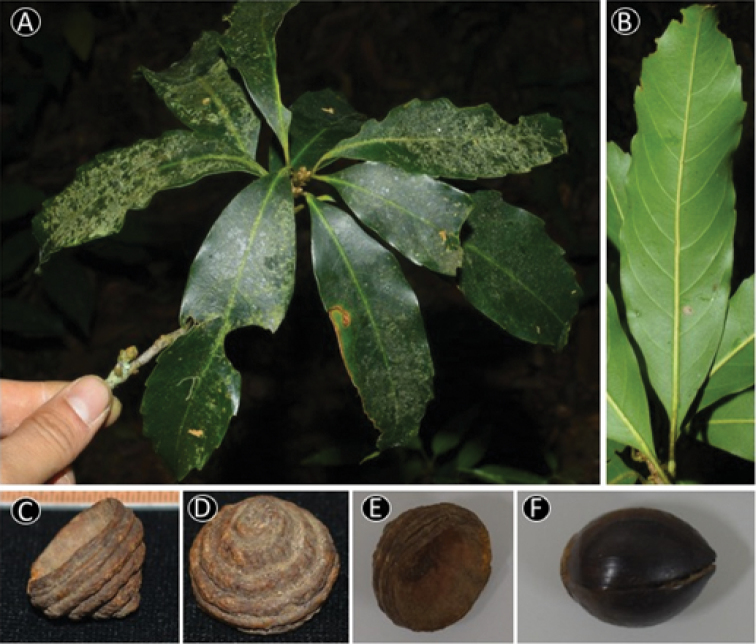
*Quercus bidoupensis* Binh & Ngoc. **A** Leafy twig **B** Abaxial side of mature leaf **C, D** Side view and base view of the cupule, respectively **E** Inside of cupule **F** Nut. Materials: **A–F** from *Tagane et al. V4328*.

#### Type.

VIETNAM. Lam Dong Province: Bidoup-Nui Ba National Park, hill evergreen forest dominated by the species of Fagaceae, 12°09'52.95"N, 108°32'00.38"E, alt. 1,698 m, 24 Feb. 2016, *Tagane S.*, *Toyama H., Nagamasu H., Naiki A., Dang Son, Nguyen V. Ngoc, Wai J*. *V4328* (holotype: KYO!; isotypes: DLU!, the herbarium of Bidoup-Nui Ba National Park).

#### Description.

Tree, 8 m tall. Buds oblong to ellipsoid, ca. 2–4 mm high, ca. 1–2 mm in diam., scales 6–7 rows, imbricate, ovate-riangular, ca. 3 × 2.5 mm, apex obtuse, margin ciliate, densely hairy or glabrous outside, glabrous inside. Twigs greyish, glabrous, lenticellate. Leaf blades oblong-lanceolate, (7.5–)10–13 × 2.5–4 cm, thinly coriaceous, blackish-brown adaxially, pale brown abaxially when dry, glabrous on both surfaces, acuminate at apex, cuneate at base, margin undulate, distinctly serrate in the upper 1/2; midrib sunken adaxially, prominent abaxially, lateral veins 10–13 pairs, slightly prominent adaxially, prominent abaxially, at an angle of 45–50 degrees from midrib and running into the teeth of margin, tertiary veins scalariform-reticulate, slightly prominent, visible on both surfaces; petioles 1.3–2 cm long, blackish when dry, glabrous. Male and female inflorescences and infructescences not seen. Fruits 2.6 cm high (including cupule); cupules obconical, 1.3–1.5 cm high, 1.3–1.7 cm in diam., enclosing 1/3 of nut when mature, outside tomentose with whitish hairs to glabrous, inside villous with erect whitish hairs, wall ca. 2–3 mm thick, bracts arranged in 5–6 rings, margin of rings entire (without scale-like structure); nut ovoid, 2.2 cm high, 1.4 cm in diam., blackish, apex acute, basal scar 0.9 cm in diam., convex, glabrous. Fruits characters were obtained from the fallen materials.

#### Phenology.

Unknown. Fallen fruits were collected in February.

#### Distribution and habitat.

VIETNAM. Lam Dong Province: Bidoup-Nui Ba National Park. At present, this species is known only from the type locality.

#### Additional specimens examined.

Vietnam. Lam Dong Province, Lan Tranh, 12°04'08.5’’N, 108°21'55.5’’E, alt. 1,695 m, 18 June 2015, *N. Nguyen, D. Luong, B. Hoang V3202* (DLU, FU).

#### Etymology.

The specific epithet “*bidoupensis*” is derived from its type locality.

### Quercus blaoensis

Taxon classificationPlantaeFagalesFagaceae

A.Camus

Fig. 8

Quercus blaoensis A.Camus, Chênes Atlas 2: 121, pl. 229 (1935–1936), nom. nud.; Les Chênes 1: 293 (1935).

#### Type.

VIETNAM. “Station agricole de Blao, province du haut Donaï”, 800 m, 25 Apr. 1933, *E. Poilane 22372* (lectotype: P [P00754000!]; isolectotypes: P [P00753999!], K [K000832201, K000832202, K000832203, K000832204, image!], G [G00358072, image!], designated here).

**Figure 8.**
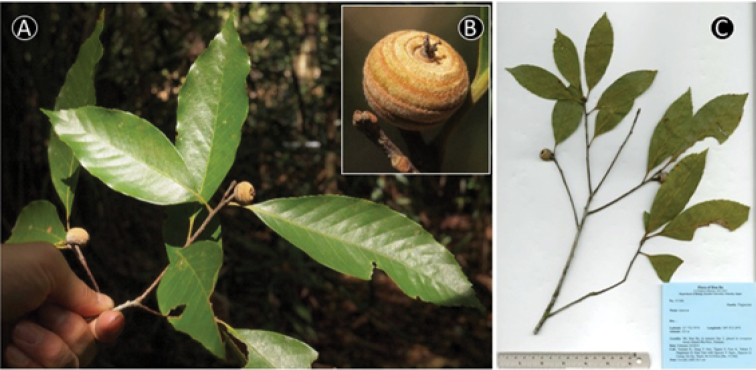
*Quercus blaoensis* A.Camus **A.** Branch with fruits **B** Young fruit **C** Dried specimen Materials: **A–C** from *Toyama et al. V1366*.

#### Distribution and habitat.

VIETNAM. Khanh Hoa Province: Hon Ba Nature Reserve. This species was found in hill evergreen forest at 225 m and 1067 m altitude.

#### Additional specimens examined.

VIETNAM. Khanh Hoa Province: Hon Ba Nature Reserve, 12°07'22.79"N, 109°00'13.29"E, alt. 225 m, 24 Feb. 2014, *Toyama H., Dang V Son, Tagane S., Fuse K., Yahara T., Nagamasu H., Hop Tran V1366* (FU, VNM, the herbarium of Hon Ba Nature Reserve); Kon Tum Province: Ngoc Linh Nature Reserve, 15°10'05.7"N, 107°45'23.6"E, alt. 1067 m, 11 Feb. 2017, *Tagane S., Nagamasu H., Nguyen N., Hoang B., Hoang S., Yang CJ. V6136* (DLU, FU, the herbarium of Ngoc Linh Nature Reserve).

#### Note.

Camus (1935) described *Quercus blaoensis* based on the specimen *Poilane 22372* from Vietnam. The authors examined specimens of *Poilane 22372* in P ([P00754000], [P00753999]) and the digitised images of the specimens in K ([K000832201, K000832202, K000832203, K000832204]) and G [G00358072]. Amongst them, only two specimens in P are fertile and only P00754000 represents diagnostic traits of a nut with cupule. Thus, the specimen *Poilane 2237*2 deposited in P [P00754000] was selected as the lectotype for *Q. blaoensis*.

### Quercus cambodiensis

Taxon classificationPlantaeFagalesFagaceae

Hickel & A.Camus

Fig. 9

Quercus cambodiensis Hickel & A.Camus, Bull. Mus. Natl. Hist. Nat. 29: 600 (1923); [P. H. Lecomte et al.] Fl. Indo-Chine 5: 946 (1929). Quercus auricoma auct. non A.Camus; Tagane et al., Tree Fl. Bokor National Park: 277 (2017).

#### Type.

CAMBODIA. ‘‘Mont. De Elephant, sol argileux tourbeu’’, 1,000 m, 6 Aug. 1919, *E. Poilane 215* (lectotype: P [P00379257!]; isolectotypes: P [P00379258!], NY [00253790, image!], designated here).

**Figure 9.**
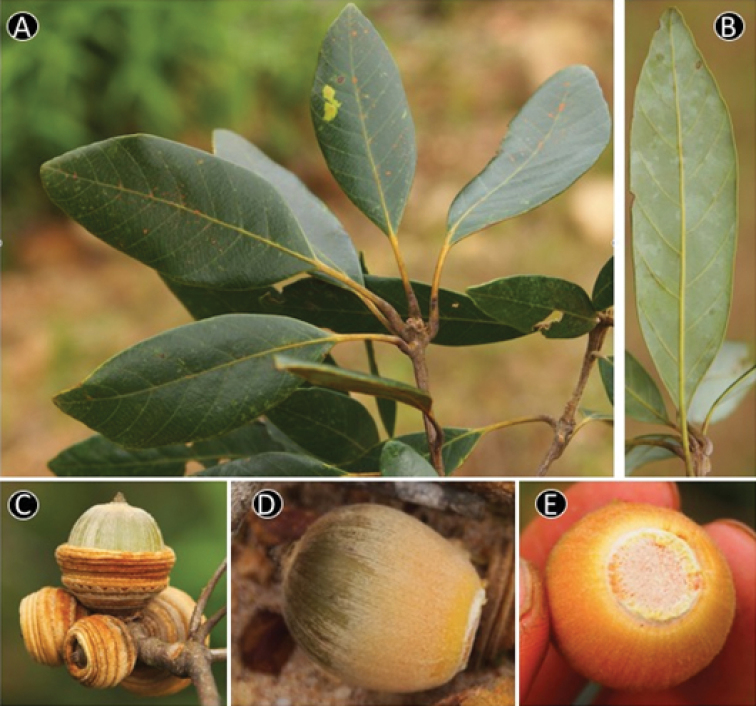
*Quercus cambodiensis* Hickel & A.Camus. **A** Leafy twig **B** Abaxial side of mature leaf **C** Infructescence and fruits **D** Nut **E** Basal scar of the nut. Materials: **A–E** from *Tagane et al. 4302*.

#### Distribution and habitat.

CAMBODIA. Kampot Province, Bokor National Park. *Quercus cambodiensis* is common in moist evergreen forest on the top plateau of Mt. Bokor.

#### Additional specimens examined.

CAMBODIA. Kampot Province, Bokor National Park: 10°37'32.30"N, 104°05'15.84"E, alt. 844 m, 17 Oct. 2012, *Tagane S., Fuse K., Choeung HN. C4302* [fr.] (FU, the herbarium of Forest Administration of Cambodia); 10°37'15.48"N, 104°05'10.71"E, 888 m, 9 Dec. 2011, *Toyama H., Tagane S., Ide T., Phourin C., Nagamasu H., Yahara T. 1834* [fr.] (FU, the herbarium of Forest Administration of Cambodia); 10°38'12.59"N, 104°02'06.37"E , 1014 m, 4 Dec. 2011, *Toyama H., Tagane S., Kajisa T., Sakata K., Nobayashi M., Mihara N., Ide T., Chhang P., Nagamasu H. 1458 & 1541* (FU, the herbarium of Forest Administration of Cambodia); ; 10°37'16.77"N , 104°01'52.32"E , 1043 m, 17 Dec. 2013, *Toyama H., Fuse K., Iwanaga F., Rueangruea S., Suddee S., Kim W., Loth M. 6276* (FU, the herbarium of Forest Administration of Cambodia); 10°38'12.59"N, 104°02'06.37"E, 1000 m, 12 Dec. 2013, *Fuse K., Suddee S., Rueangruea S., Iwanaga F., Loth* M. *Fuse K. 6342* (FU, the herbarium of Forest Administration of Cambodia).

#### Note.

Hickel and Camus (1923) described *Quercus cambodiensis* Hickel & A.Camus based on two specimens collected by E. Poilane (*Poilane 215 and Poilane 270*) from Cambodia. Deng et al. (2010) selected *Poilane 215* as the lectotype for *Q. cambodiensis*. However, there are three specimens of *Poilanei 215* in P [P00379257, P00379258] and NY [00253790], amongst which only one specimen [P00379257] represents the diagnostic traits of nuts and cupules. Thus, specimen [P00379257] was selected as the lectotype of *Q. cambodiensis*.

### Quercus camusiae

Taxon classificationPlantaeFagalesFagaceae

Trel. ex Hickel & A.Camus

Fig. 10

Quercus camusiae Trel. ex Hickel & A.Camus, Fl. Indo-Chine 5: 957 (1929). Quercus geminata Hickel & A.Camus, Bull. Mus. Natl. Hist. Nat.: 599 (1923), nom. illegit. Cyclobalanopsis camusiae (Trel. ex Hickel & A.Camus) Y.C.Hsu & H.W.Jen, J. Beijing Forest. Univ. 15(4): 44 (1993).

#### Type.

VIETNAM. Annam [Trung Ky]: ‘‘Pres de Nha-trang, massif de Honba,’’ 1,000–1,500 m, 18–20 Sep. 1918, *A. Chevalier 38650* (lectotype: P [P00379252!]; isolectotype: P [P00379253!], designated here).

**Figure 10.**
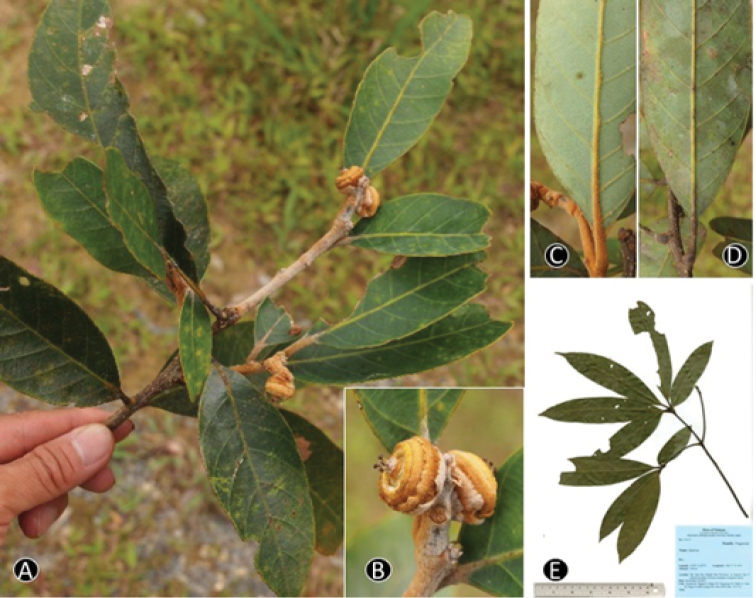
*Quercus camusiae* Trel. ex Hickel & A.Camus. **A** Branch with young fruit, **B.** Infructescence and young fruits **C, D** Abaxial side of young and mature leaf, **E.** Dried specimen. Materials: **A–D** from *Tagane et al. V342* **E** from *Toyama et al. V2173*.

#### Distribution and habitat.

VIETNAM. Khanh Hoa Province: Hon Ba Nature Reserve. *Quercus camusiae* was found on slope of lower montane evergreen forest at 995 m, 1336 m and 1498 m altitude.

#### Additional specimens examined.

VIETNAM. Khanh Hoa Province, Hon Ba Nature Reserve, 12°07'08.64"N, 108°56'51.99"E, alt. 1,498 m, 18 July 2013, *Tagane S., Yahara T., Nagamasu H., Fuse K., Toyama H., Tran H., Son VD. V290* (FU, the herbarium of Hon Ba NR, VNM); 12°07'10.02"N, 108°56'51.71"E, alt. 995 m, 19 July 2013, *Tagane S., Yahara T., Nagamasu H., Fuse K., Toyama H., Tran H., Son VD. V342* [young fr.] (FU, the herbarium of Hon Ba NR, VNM); 12°07'11.42"N, 108°57'25.76"E, alt. 1336 m, 25 Nov. 2014, *Toyama H., Tagane S., Dang VS., Nagamasu H., Naiki A., Tran H., Yang CJ. V2173* (FU, VNM, the herbarium of Hon Ba Nature Reserve).

#### Note.

*Quercus camusiae* Trel. ex Hickel & A.Camus was described by Hickel and Camus (1929) based on a specimen collected by A. Chevalier from Vietnam (“Annam: Pres de Nha-trang, massif de Honba”) to replace the name *Q. geminate* Hickel & Camus (1923) without any collection number. Later, Camus (1938) cited specimen *Chevalier 38650* from Vietnam as the type specimen of *Q. camusiae*. Two specimens of *Chevalier 38650* were found in P comprising [P00379252] and [P00379253], between which only one specimen [P00379252] represents the diagnostic traits of nuts and cupules. Thus, specimen [P00379252] was selected as the lectotype of *Q. camusiae.*

### Quercus dilacerata

Taxon classificationPlantaeFagalesFagaceae

Hickel & A.Camus

Quercus dilacerata Hickel & A.Camus, [P. H. Lecomte et al.] Fl. Indo-Chine 5: 960 (1929).

#### Type.

VIETNAM. “Tonkin: Km. 8 du col de Lo qui Ho près de Chapa”, 1800 m, 29 July 1926, *E. Poilane 12645* (lectotype: P [P00753996!]; isolectotype: P [P00753997!], designated here).

#### Distribution and habitat.

VIETNAM. Lao Cai Province: Lo Qui Ho Pass, Chapa.

#### Note.

In the original publication of *Quercus dilacerata*, Hickel and Camus (1929) cited the specimen collected by E. Poilane from Tonkin, Km. 8 du col de Lo qui Ho près de Chapa, Vietnam without any collection number. Two specimens of *Quercus* collected by Poilane from Tonkin, Km. 8 du col de Lo qui Ho près de Chapa, Vietnam were found in P with collector’s number *12645* (P [P00753996], [P00753997]). Both specimens are fertile and consistent with the description of Hickel and Camus (1929). Here, the specimen [P00753996] with more nuts was designated as the lectotype for *Q. dilacerata.*

### Quercus donnaiensis

Taxon classificationPlantaeFagalesFagaceae

A.Camus

Fig. 11

Quercus donnaiensis A.Camus, Chênes Atlas 2: 119, pl. 227 (1935–1936), nom. nud.; Les Chênes 1: 190 (1935).

#### Type.

VIETNAM. “Annam: Près de Sapoum, près station agricole de Blao, prov. du Haut Donai”, 1000–1100 m, 9 Jan. 1935, *E. Poilanei 23732* (lectotype: P [P00753995!]; isolectotype: P [P00753994!], designated here).

**Figure 11.**
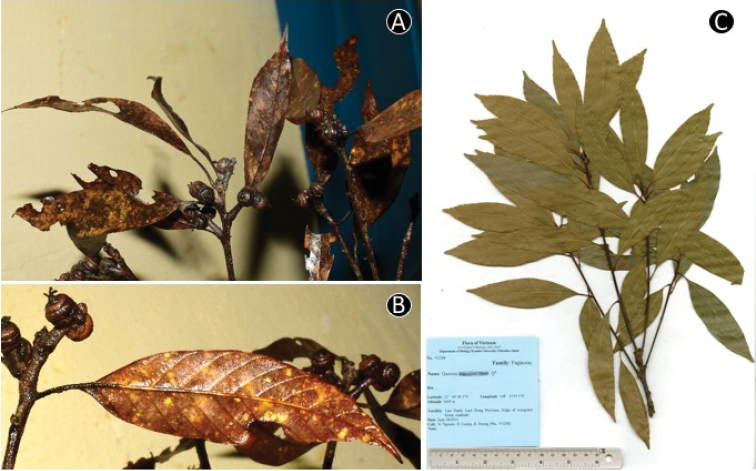
*Quercus donnaiensis* A.Camus. **A** Leafy twig **B** Infructescence, young fruits and abaxial side of mature leaf **C** Dried specimen. Materials: **A, B** from *Tagane S., Wai J. V4398* **C** from *Ngoc et al. V3208*.

#### Distribution and habitat.

VIETNAM. Lam Dong Province: Lan Tranh wards. In this study, *Q. donnaiensis* was found in lower montane evergreen forest, beside a stream at 1489 m altitude and at the edge of evergreen forest, at 1695 m altitude.

#### Additional specimens examined.

VIETNAM. Lam Dong Province, Lan Tranh wards, 12°04'08.5"N, 108°21'55.5"E, alt. 1,695 m, 18 June 2015, *N. Nguyen, D. Luong, B. Hoang V3208* (DLU, FU); Lam Dong Province, Bi Doup-Nui Ba National Park, 12°11'19.8"N, 108°40'48.3"E, alt. 1,489 m, 25 Feb. 2016, *Tagane S., Wai J. V4398* (FU, DLU, the herbarium of Bidoup-Nui Ba National Park).

#### Note.

*Quercus donnaiensis* was described by Camus (1935), based on the specimen *Poilanei 23732* collected from Vietnam and then illustrated by Camus (1935–1936). Two specimens of *Poilanei 23732* deposited in P have acorns and are consistent with the illustration and description of Camus (1935a, b). Amongst them, the specimen *Poilane 23732* in P [P00753995] with nuts, as it better represents the diagnostic traits, was designated as the lectotype of *Q. donnaiensis.*

### Quercus honbaensis

Taxon classificationPlantaeFagalesFagaceae

Binh, Tagane & Yahara sp. nov.

urn:lsid:ipni.org:names:77175740-1

Fig. 12

#### Diagnosis.

*Quercus honbaensis* is distinguished from *Q. langbianensis* *s. str.* in having shorter petiole of 0.8–1 cm long (vs. 1–1.8 cm long), more secondary veins ((10–)14–16 pairs vs. 10–12 pairs) and obconical cupules (vs. cup-shaped).

**Figure 12.**
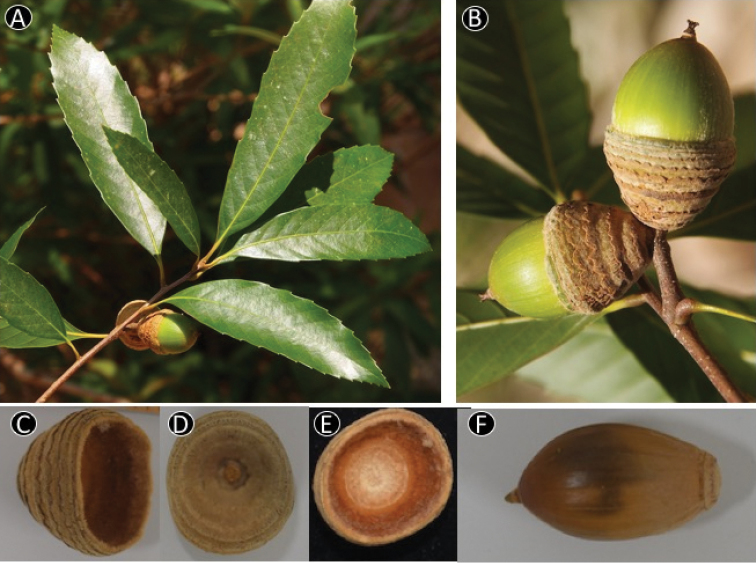
*Quercus honbaensis* Binh, Tagane & Yahara. **A** Leafy twig **B** Infructescence and mature fruits, **C, D** Side view and base view of the cupule, respectively, **E.** Inside of cupule, **F.** Nut. Materials: **A–F** from *Toyama et al. V1378*.

#### Type.

VIETNAM. Khanh Hoa Province: Hon Ba Nature Reserve, evergreen forest along river, 12°07'22.79"N, 109°00'13.29"E, alt. 225 m, 24 Feb. 2014, *Toyama H., Dang V Son., Tagane S., Fuse K., Yahara T., Nagamasu H., Hop Tran V1378* (holotype: KYO!; isotypes: FU!, VNM!).

#### Description.

Tree, 12 m tall. Buds ovoid, ca. 3–4 mm high, ca. 2–3 mm in diam., scales in 4–6 rows, imbricate, ovate-triangular, ca. 1 × 1.5 mm, apex obtuse, margin yellowish-brown ciliate, appressed hairy on both surfaces. Young twigs greyish-brown, 1–1.2 mm in diam., curly hairy, sometimes sulcate, old twigs greyish-brown, glabrous, lenticellate. Leaf blades lanceolate to oblanceolate, (3.6–)11–16.5 × (1.4–)2–5.2 cm, acute at apex, cuneate at base, margin regularly distinctly serrate in the upper 5/6–3/4(–2/3), glabrous on both surfaces; midrib slightly prominent adaxially, prominent abaxially, lateral veins (9–)10–14(–16) pairs, straight and running into the teeth of margin, slightly prominent adaxially, prominent abaxially, at an angle of 40–45 degrees from midrib, tertiary veins scalariform-reticulate, faintly visible on both surfaces; petioles 0.8–1 cm long, tomentose when young, soon glabrous. Male and female inflorescences not seen. Infructescences axillary, erect, rachis 0.5–1.4 cm long, 1–2 mm in diam., glabrous. Mature fruits 2–3.5 cm high (including cupule), usually 1 (or 2) per infructescence, sessile; cupules obconical, 1.4–1.6 cm high, 1.5–1.8 cm in diam., enclosing 1/3–1/2 of the nut, wall covered with densely whitish- to yellowish-brown hairs, bracts arranged in 6–8 rings, margin of the ring undulate; nut obovoid to ellipsoid, 2.3–2.8 cm high, 1.3–1.7 cm in diam., apex obtuse, sparsely hairy except densely appressed hairy around stylopodia and basal scar, stylopodia up to 3 mm long, basal scar 0.7–0.8 cm in diam., convex.

#### Phenology.

Fruiting specimens were collected in February.

#### Distribution and habitat.

VIETNAM. Khanh Hoa Province: Hon Ba Nature Reserve. This species is known only from the type locality. A few individuals were found in evergreen forest from 225–617 m elevation.

#### Additional specimens examined.

VIETNAM. Khanh Hoa Province: Hon Ba Nature Reserve, 12°06'33.41"N, 108°59'24.89"E, alt. 367 m, 22 July 2013, *Tagane S., Yahara T., Nagamasu H., Fuse K., Toyama H., Tran H., Dang V.S., V744* (FU, VNM, the herbarium of Hon Ba NR); 12°06'39.77"N, 108°58'59.23"E, alt. 617 m, 22 Feb. 2014, *Toyama H., Dang V.S., Tagane S., Fuse K., Yahara T., Nagamasu H., Tran H., Nguyen V.N., Nguyen Q.C., Do N.T., Ho N.P.H., V1200* (FU, VNM, the herbarium of Hon Ba NR); 12°06'31.2"N, 108°59'14.1"E, alt. 400 m, 13 July 2014, *Tagane S., Kanemitsu H., Dang V.S., Tran H., Hanh N., Loi X.N., Thach N.D., Dinh N., Hieu P.N.H., V1548* (FU, VNM, the herbarium of Hon Ba NR); 12°07'22.79"N, 109°00'13.29"E, alt. 225 m, 15 July 2014, *Tagane S., Kanemitsu H., Dang V.S., Tran H., Loi X.N., Thach N.D., Dinh N., Hieu P.N.H., V1662* (FU, VNM, the herbarium of Hon Ba NR).

#### Etymology.

The specific epithet “*honbaensis*” is derived from its type locality, Mt. Hon Ba.

### Quercus langbianensis

Taxon classificationPlantaeFagalesFagaceae

Hickel & A.Camus

Fig. 13

Quercus langbianensis Hickel & A.Camus, Ann. Sci. Nat., Bot. 10, 3: 382 (1921); [P.H. Lecomte et al.] Fl. Indo-Chine 5: 950 (1929).

#### Type.

VIETNAM. “Annam: massif du Lang-Bian, grand Piton Lang-Bian, près du village de Beneur”, 1500–2000 m, 15 Feb. 1914, *A.J.B Chevalier 30029* (holotype: P [P00379254!]; isotypes: P [P00379255! P00379256!]).

**Figure 13.**
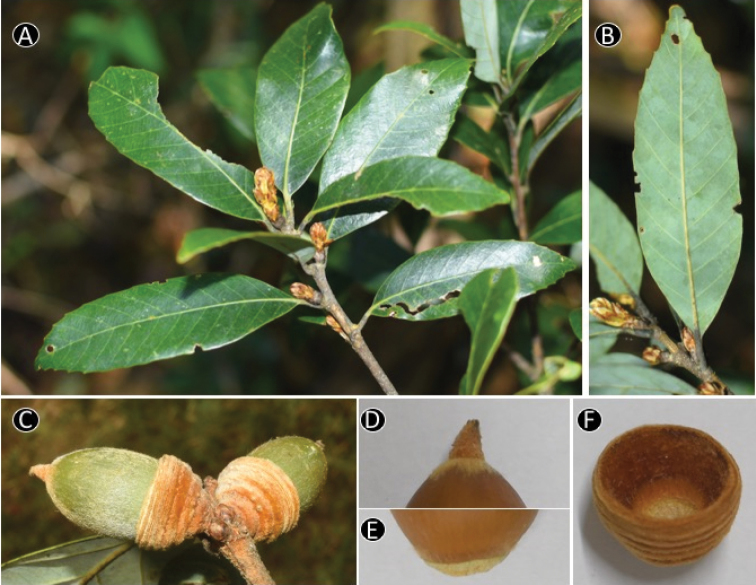
*Quercus langbianensis* Hickel & A.Camus. **A** Leafy twig **B** Abaxial side of mature leaf **C** Infructescence and mature fruits **D** Apex of the nut **E** Basal scar of the nut **F** Inside of cupule. Materials: **A, B** from *Tagane et al. V 4165* **C–F** from *Tagane et al. V4166*.

#### Distribution and habitat.

VIETNAM. Lam Dong Province: Bidoup-Nui Ba National Park. In this study, *Quercus langbianensis* *s. str.* is known only from Lang-Bian Mountain of the national park, in lower montane evergreen forest at 1472 m and 1533 m altitude.

#### Additional specimen examined.

VIETNAM. Bidoup-Nui Ba National Park, 12°10'34.04"N, 108°40'28.93"E, alt. 1,472 m, 29 Feb. 2016, *Tagane S.*, *Son VD., Wai J. V4465* (DLU, FU, the herbarium of Bidoup-Nui Ba National Park); ibid. 12°10'34.09"N, 108°41'04"E, alt. 1,533 m, 22 Feb. 2016, *Tagane S.*, *Nagamasu H., Naiki A., Dang V. Son, Nguyen V. Ngoc., Binh T. Hoang V4165, V4166* (DLU, FU, the herbarium of Bidoup-Nui Ba National Park); 12°10'34.7"N, 108°41'08.4"E, alt. 1,533 m, 21 Feb. 2016, *Tagane S.*, *Nagamasu H., Naiki A., Dang V. Son, Nguyen V. Ngoc., Binh T. Hoang V3962* (DLU, FU, the herbarium of Bidoup-Nui Ba National Park).

## Supplementary Material

XML Treatment for Quercus baniensis

XML Treatment for Quercus baolamensis

XML Treatment for Quercus bidoupensis

XML Treatment for Quercus blaoensis

XML Treatment for Quercus cambodiensis

XML Treatment for Quercus camusiae

XML Treatment for Quercus dilacerata

XML Treatment for Quercus donnaiensis

XML Treatment for Quercus honbaensis

XML Treatment for Quercus langbianensis

## Appendix 1

**Table:** Voucher information and GenBank accession numbers for samples used in this study (newly sequenced data).

Species	Vouchers	Elevation	GenBank accession no.			MIG-seq
			*rbc*L	*mat*K	ITS	
*Quercus annulata*	*Tagane et al. V4730*	1850 m	LC318796	LC318516	MF770291	+
*Quercus auricoma*	*Ngoc et al. V3135*	518 m	LC318778	LC318498	MF770277	+
*Quercus auricoma*	*Ngoc et al. V3138*	556 m	LC318779	LC318499	–	+
*Quercus austrocochinchinensis*	*Ngoc et al. V3129*	249 m	LC318777	LC318497	MF770276	+
*Quercus austrocochinchinensis*	*Ngoc et al. V3156*	340 m	LC318780	LC318500	MF770278	+
*Quercus baniensis*	*Tagane et al. V3089*	707 m	LC318775	LC318495	MF770274	+
*Quercus baniensis*	*Hoang & Tagane V6922*	789 m	LC318802	LC318522	MF770296	+
*Quercus baolamensis*	*Ngoc et al. V3191*	1000 m	LC318782	LC318502	MF770280	+
*Quercus bidoupensis*	*Ngoc et al. V3202*	1695 m	LC318783	LC318503	–	–
*Quercus bidoupensis*	*Tagane et al. V4328*	1698 m	LC318793	LC318513	MF770288	+
*Quercus blaoensis*	*Toyama. et al. V1366*	225 m	LC318768	LC318488	MF770269	+
*Quercus blaoensis*	*Tagane et al. V6136*	1067 m	LC318799	LC318519	–	+
*Quercus braianensis*	*Ngoc et al. V3219*	1535 m	LC318785	LC318505	MF770281	+
*Quercus braianensis*	*Ngoc et al. V4445*	1464 m	LC318794	LC318514	MF770289	+
*Quercus cambodiensis*	*Tagane et al. 1834*	888 m	–	–	–	–
*Quercus cambodiensis*	*Tagane et al. 1458*	1014 m	–	–	–	–
*Quercus cambodiensis*	*Tagane et al. 1541*	1014 m	–	–	–	–
*Quercus cambodiensis*	*Tagane et al. 6276*	1043 m	–	–	–	–
*Quercus cambodiensis*	*Tagane et al. 6342*	1000 m	–	–	–	–
*Quercus cambodiensis*	*Tagane et al. 4302*	844 m	LC318766	LC318445	MF770268	+
*Quercus camusiae*	*Tagane et al. V290*	1498 m	–	–	–	–
*Quercus camusiae*	*Tagane et al. V342*	1511 m	LC318787	LC318507	–	–
*Quercus camusiae*	*Toyama. et al. V2173*	1336 m	LC318773	LC318493	MF770272	+
*Quercus donnaiensis*	*Ngoc et al. V3208*	1695 m	LC318784	LC318504	–	+
*Quercus donnaiensis*	*Tagane & Wai 4398*	1489 m	–	–	–	–
*Quercus djiringensis*	*Dung et al. V5537*	N/A	LC318797	LC318517	MF770292	+
*Quercus djiringensis*	*Dung et al. V5538*	N/A	LC318798	LC318518	MF770293	+
*Quercus helferiana*	*Ngoc et al. V3169*	1400 m	LC318781	LC318501	MF770279	+
*Quercus helferiana*	*Ngoc et al. V3244*	1580 m	LC318786	LC318506	MF770282	+
*Quercus honbaensis*	*Tagane et al. V744*	367 m	–	–	–	–
*Quercus honbaensis*	*Toyama. et al. V1200*	617 m	LC318767	LC318446	–	–
*Quercus honbaensis*	*Tagane. et al. V1548*	400 m	LC318770	LC318490	–	–
*Quercus honbaensis*	*Tagane. et al. V1662*	225 m	LC318771	LC318491	–	–
*Quercus honbaensis*	*Toyama. et al. V1378*	225 m	LC318769	LC318489	MF770270	+
*Quercus kerrii*	*Tagane et al. V6765*	833 m	LC318801	LC318521	MF770295	+
*Quercus langbiangensis*	*Tagane et al. V3962*	1533 m	LC318790	LC318510	MF770285	+
*Quercus langbiangensis*	*Tagane et al. V4165*	1533 m	LC318791	LC318511	MF770286	+
*Quercus langbiangensis*	*Tagane et al. V4166*	1533 m	LC318792	LC318512	MF770287	+
*Quercus langbiangensis*	*Tagane et al. V4465*	1472 m	LC318795	LC318515	MF770290	+
*Quercus macrocalyx*	*Tagane et al. V6457*	1376 m	LC318800	LC318520	MF770294	+
*Quercus neglecta*	*Ngoc et al. V3587*	546 m	LC318788	LC318508	MF770283	+
*Quercus neglecta*	*Ngoc et al. V3788*	1062 m	LC318789	LC318509	MF770284	+
*Quercus poilanei*	*Toyama. et al. V1895*	1644 m	LC318772	LC318492	MF770271	+
*Quercus poilanei*	*Yahara et al. V2986*	1412 m	LC318774	LC318494	MF770273	+
*Quercus poilanei*	*Tagane et al. V3113*	1310 m	LC318776	LC318496	MF770275	+
*Trigonobalanus verticillata* (Outgroup)	*Ngoc et al. V5764*	1735 m	LC318965^*^	LC318549^*^	MF770380^*^	+

(+): Used for analyses in this paper; (–): Not used for analyses in this paper. (*) From GenBank.

## Supplementary materials

Supplementary material 1 Figure S1, S2 Data type: Phylogenetic trees Explanation note: Figure S1. Bayesian phylogeny of 29 samples of *Quercus* and one *Trigonobalanus* (outgroup) based on ITS sequences. Branches are labeled with posterior probabilites. Figure S2. Bayesian phylogeny of 29 samples of *Quercus* and one *Trigonobalanus* (outgroup) based on concatenated *rbc*L and *mat*K sequences. Branches are labeled with posterior probabilities. This is an open access article distributed under the terms of the Creative Commons Attribution License (CC BY 4.0), which permits unrestricted use, distribution, and reproduction in any medium, provided the original author and source are credited.Hoang Thi Binh, Nguyen Van Ngoc, Shuichiro Tagane, Hironori Toyama, Keiko Mase, Chika Mitsuyuki, Joeri Sergej Strijk, Yoshihisa Suyama, Tetsukazu Yahara
