# Supplementary material for: A taxonomic study of Quercus langbianensis complex based on morphology and DNA barcodes of classic and next generation sequences
Source: PhytoKeys. 2018 Feb 7;(95):37–70. doi: 10.3897/phytokeys.95.21126 (PMC5904329; doi:10.3897/phytokeys.95.21126)
Supplement: Supplementary material 1 — Figure S1, S2 [file phytokeys-95-037-s001.pdf]

Supplement

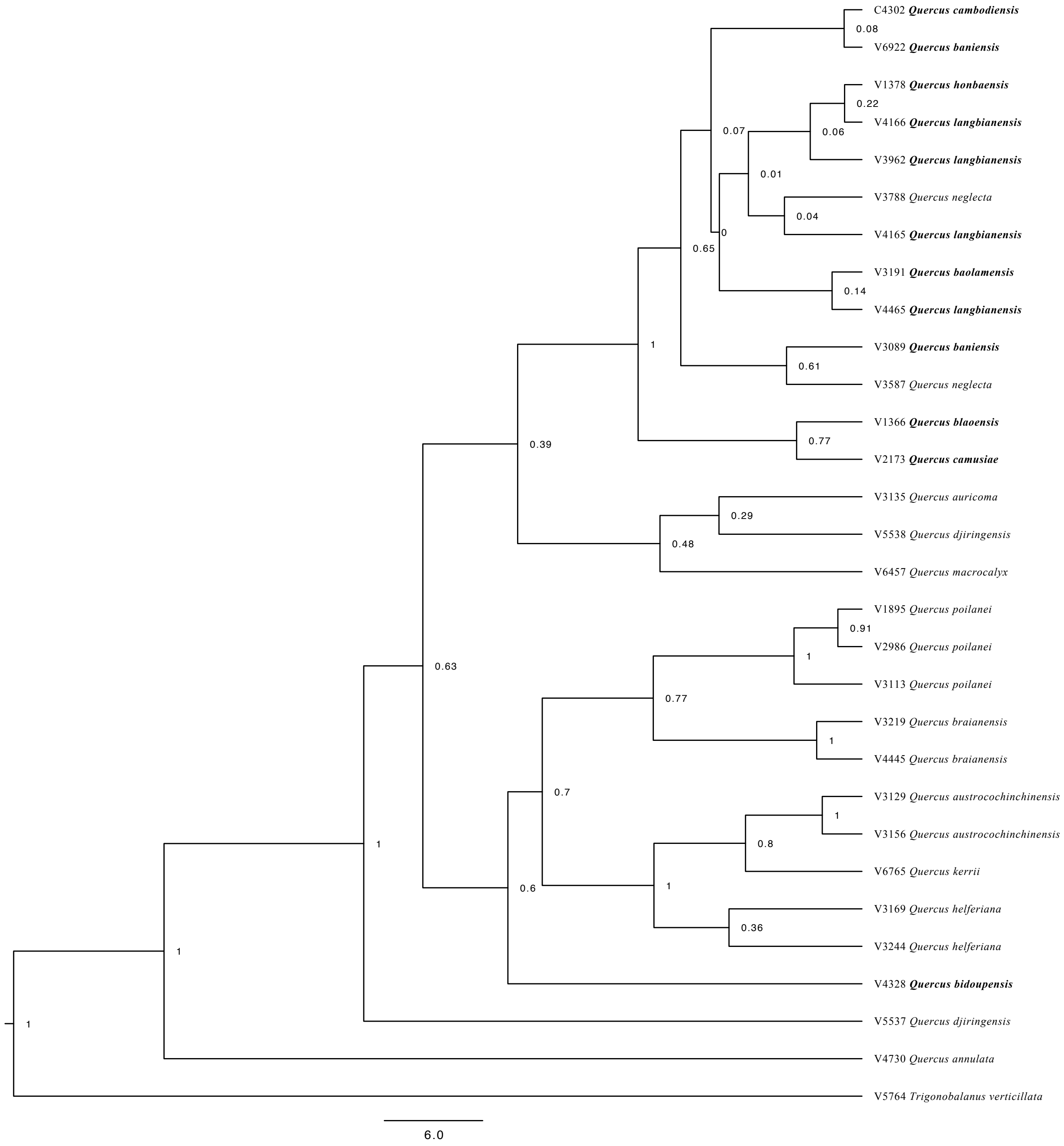

Supplementary *Figure S1:* Bayesian phylogeny of 29 samples of *Quercus* and one *Trigonobalanus* (outgroup) based on ITS sequences. Braches are labeled with posterior probabilities.

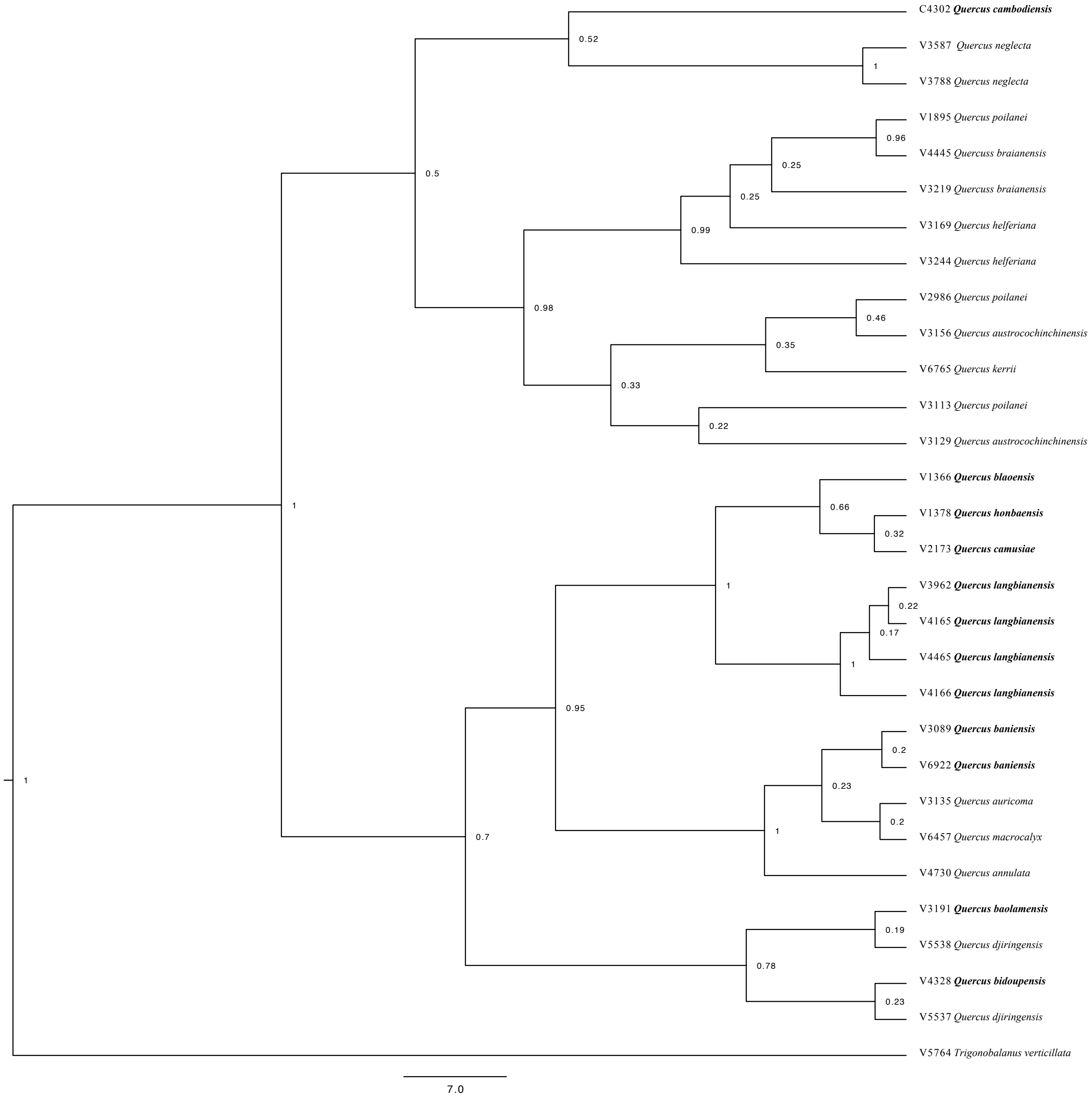

Supplementary ***Figure S2:*** Bayesian phylogeny of 29 samples of *Quercus* and one *Trigonobalanus* (outgroup) based on concatenated *rbc*L and *mat*K sequences. Braches are labeled with posterior probabilities.
